# Genomic structure and insertion sites of *Helicobacter pylori* prophages from various geographical origins

**DOI:** 10.1038/srep42471

**Published:** 2017-02-16

**Authors:** Filipa F. Vale, Alexandra Nunes, Mónica Oleastro, João P. Gomes, Daniel A. Sampaio, Raquel Rocha, Jorge M. B. Vítor, Lars Engstrand, Ben Pascoe, Elvire Berthenet, Samuel K. Sheppard, Matthew D. Hitchings, Francis Mégraud, Jamuna Vadivelu, Philippe Lehours

**Affiliations:** 1 Host-Pathogen Interactions Unit, Research Institute for Medicines (iMed-ULisboa), Faculdade de Farmácia da Universidade de Lisboa, Lisboa, Portugal; 2 Université de Bordeaux, Centre National de Référence des Campylobacters et Hélicobacters, Bordeaux, France; 3 Bioinformatics Unit, Department of Infectious Diseases, National Institute of Health, Lisboa, Portugal; 4 National Reference Laboratory of Gastrointestinal Infections, Department of Infectious Diseases, National Institute of Health, Lisboa, Portugal; 5 Innovation and Tecnhology Unit, Department of Human Genetics, National Institute of Health, Lisboa, Portugal; 6 Department of Biochemistry and Human Biology, Faculdade de Farmácia, Universidade de Lisboa, Lisboa, Portugal; 7 Department of Microbiology, Tumor and Cell Biology, Karolinska Institute, Stockholm, Sweden; 8 The Milner Centre for Evolution, Department of Biology and Biochemistry, University of Bath, Bath, UK; 9 Institute of Life Science, Swansea University Medical School, Swansea, UK; 10 UM Marshall Centre and Department of Medical Microbiology, University of Malaysia, Kuala Lumpur, Malaysia; 11 INSERM U1053, Bordeaux, France

## Abstract

*Helicobacter pylori* genetic diversity is known to be influenced by mobile genomic elements. Here we focused on prophages, the least characterized mobile elements of *H. pylori*. We present the full genomic sequences, insertion sites and phylogenetic analysis of 28 prophages found in *H. pylori* isolates from patients of distinct disease types, ranging from gastritis to gastric cancer, and geographic origins, covering most continents. The genome sizes of these prophages range from 22.6–33.0 Kbp, consisting of 27–39 open reading frames. A 36.6% GC was found in prophages in contrast to 39% in *H. pylori* genome. Remarkably a conserved integration site was found in over 50% of the cases. Nearly 40% of the prophages harbored insertion sequences (IS) previously described in *H. pylori*. Tandem repeats were frequently found in the intergenic region between the prophage at the 3′ end and the bacterial gene. Furthermore, prophage genomes present a robust phylogeographic pattern, revealing four distinct clusters: one African, one Asian and two European prophage populations. Evidence of recombination was detected within the genome of some prophages, resulting in genome mosaics composed by different populations, which may yield additional *H. pylori* phenotypes.

*Helicobacter pylori* is a major widely distributed human pathogen, with one out of two persons being colonized by this bacterium. Infection by *H. pylori* is associated with gastritis and may progress to more severe conditions, including peptic ulcer and, in rare cases, gastric adenocarcinoma and gastric MALT (mucosa associated lymphoid tissue) lymphoma. *H. pylori* presents a phylogeographic distribution, reflecting a pattern of co-evolution with the human host^1^.

Genome rearrangement and high rate of mutation are characteristics of *H. pylori*^2^,^3^, described as a highly genetic diverse^4^. Furthermore, this variability is reinforced by epigenome diversity^5^,^6^. Among the factors for increased diversity there are mobile genomic elements, including the *cag*-pathogenicity island (PAI)^7^, insertion sequences^8^, restriction-modification systems^9^,^10^ and prophages^11^. Furthermore, *H. pylori* is among the most recombinogenic known human pathogens^12^.

There are about 10^31 phages on the planet, with phages exceeding bacteria in number by tenfold, but less than an estimated 1% have been described^13^. Temperate phages contribute to the evolution of most bacteria, by promoting the transduction of various genes involved in virulence, fitness, and antibiotic resistance^14^. Despite the putative bacterium–phage evolutionary conflict, phages profit from promoting the survival and proliferation of their hosts^15^. Likewise, prophages may harbor cargo genes, or “morons”, which while are not essential for the phage, benefits the host. Some very well known lysogenic phages carry genes that enhance the virulence of the bacterial host^16^. In addition, the deletion of prophages from *E. coli* revealed that prophages improved the surviving under adverse environmental conditions, including acid stress or early biofilm formation^17^. Prophages may therefore work as gene reservoirs, many of which benefit pathogens, in ways which are only just beginning to be determined^18^. In a hostile environment like the human stomach, any metabolic advantage or resistance/tolerance mechanism provided by prophages should be important in improving bacterial host competitiveness. Prophage induction may also be used as a weapon for colonizing new niches^19^, displacing native strains, although this strategy may be rarely used, first by the creation of lysogens in the susceptible population, second by the cost of cell lysis in a fraction of the population, and third due to the purifying selection of prophages^20^. Taken together, these properties may explain why prophages are more frequent in pathogenic bacteria^21^. Host-prophage driven selection and genetic flux occurs even for prophage genes that do not effect host physiology^20^. Thus, the role of prophages in disease establishment is being progressively acknowledged.

The first descriptions of *H. pylori* phages came from the observation of micrographs where particles compatible with phages are observed^22^,^23^,^24^,^25^,^26^. The development of the genomic studies, especially using high-throughput genome sequencing led to the first reports of prophages, some remnant^27^, others apparently complete and capable of going through a lytic cycle^11^,^28^,^29^,^30^,^31^,^32^. Strains carrying prophages do not appear to have a higher pathogenicity or association with particular disease patterns^11^,^33^, but it has been suggested that the presence of phage orthologous genes correlates with the presence of *cagA* and/or *vacA* virulence genes^34^. The population to which prophages belong is determined by prophage sequence typing (PST), which targets two prophage genes (integrase and holin) of *H. pylori* and applies a Bayesian clustering analysis for the identification of distinct genetic populations. Currently there are 4 prophage populations described, hpAfrica1, hpEastAsia, hpNEurope and hpSWEurope^33^. On the other hand, the bacterial population is determined by MultiLocus Sequence Typing (MLST), which is based on the analysis of 7 bacterial housekeeping genes. Presently, there are 7 seven *H. pylori* populations described, hpAfrica1, hpAfrica2, hpNEAfrica, hpSahul, hpAsia2, hpEastAsia and hpEurope^35^. Recently, using the PST method, we determined that *H. pylori* prophage genes, namely integrase and holin genes present a phylogeographic distribution. Furthermore, the European *H. pylori* population (hpEurope), which could not be discriminated using the MLST method, was separated into two different populations (hpNEurope and hpSWEurope) using these two prophage genes^33^.

The number of complete phage genomes available in GenBank is low. Despite the recent discovery of the importance of prophages in the diversity of *H. pylori*^11^, they remain poorly characterized. The lack of information on bacteriophages of *H. pylori* prompted this study. Based on the presence of the prophage integrase gene we determined that an estimated 20% of *H. pylori* strains carry prophages^11^,^33^. Based on PCR screening, we compiled a collection of *H. pylori* strains carrying prophages^33^. We therefore undertook a more holistic approach, using the next generation sequencing (NGS) method to study the full genome of strains (Whole-genome sequencing) from this collection as well *H. pylori* strains presenting prophages found in public databases. This information allowed us to identify phage sequences, which were then used for comparative genomics. Our results have increased our knowledge on *H. pylori* prophage genomic organization into syntenic blocks, insertion sites, phylogeography, and diversity. The detailed genomic structure of 28 prophages reported here will provide in the future an important basis to identify the function of prophage genes and to verify if prophages provide advantageous phenotypes.

## Results

A summary of *H. pylori* sequenced genomes can be found in Table S1 (Supplementary Information).

### Prophage genome characteristics

We were able to close the physical gaps between contigs in over 90% of the prophage genomes using PCR and Sanger sequencing. In most cases the prophage contigs were separated at insertion sequences, repetition zones and/or sequences showing homology with other bacterial genes. A prophage was considered intact if the size was larger than 20 Kb. According to this criterion, prophages were found to be intact in 23 of the 28 genomes (82%) (Table 1). The other five genomes showed remnant prophages (Table S2, Supplementary Information) between 11.6 Kb and 19.8 Kb. Intact prophages were initially divided in several contigs (min 1- max 7) and have an average of 34 predicted genes (min 24, max 39), 28.7 Kb (min 22.6, max 33.0), and 36.7% GC, which is in line with other *H. pylori* prophages described^11^,^28^. The bacterial average GC percentage was 39.0%, suggesting horizontal gene transfer of the prophage region.

**Figure 1:**
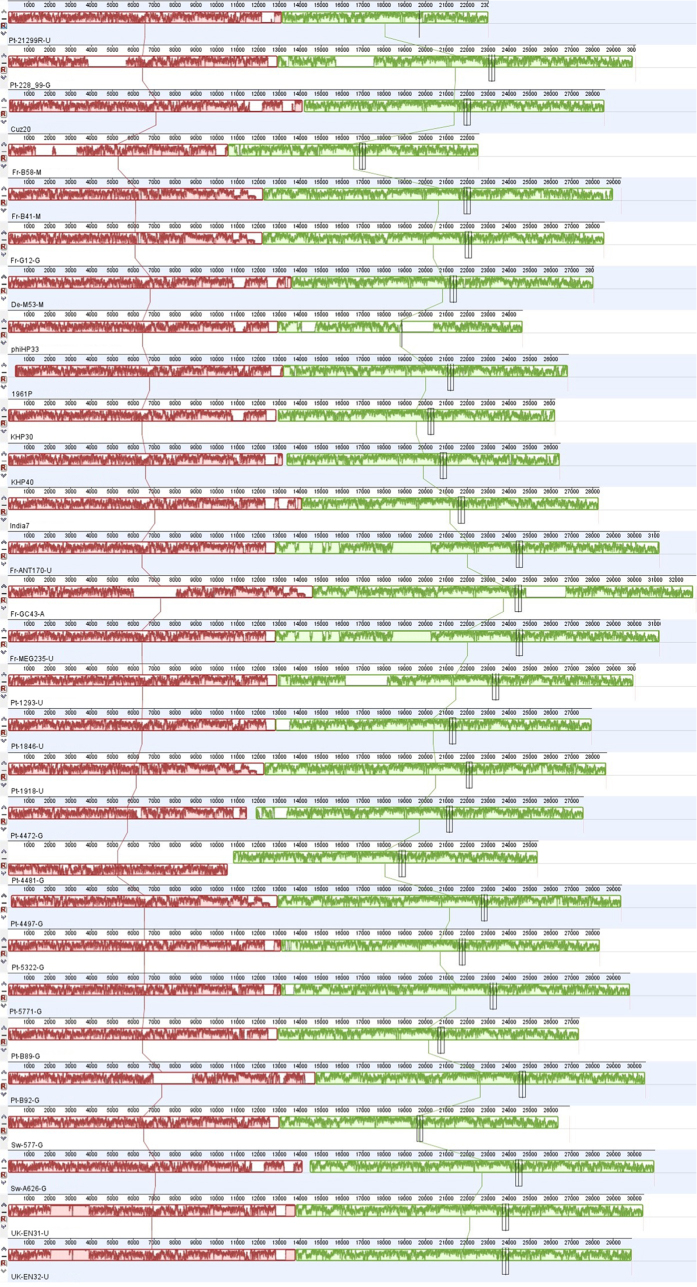
Alignment of 29 complete prophages, using Mauve software (version 2.3.1).

The gene content of intact prophages was similar to phage KHP30, a known complete phage with lytic cycle^30^. The intact prophage genomes had a rather similar sequence (Figures 1 and S1, Supplementary Information) with a reasonably conserved gene order (Tables S3 and S4, Supplementary Information) and in clear contrast with the host *H. pylori*, where the occurrence of genome rearrangement is well known^36^. Genome annotation of prophage genes produced with either RAST^37^ or PHAST^38^ revealed that most of the open reading frames (ORF) corresponded to hypothetical proteins, disclosing the diversity of prophage genes and the consequent difficulty in the annotation process. The annotation with Phages 1.0 (http://www.phantome.org/PhageSeed/Phage.cgi?page=phast) did not add more information and was not further considered.

The similarity of prophage genomes was also quantified as a heat-map (Figure S2, Supplementary Information). This similarity matrix confirmed the percentages of bases which were identical. Only one prophage genome, strain Pt-4481-G, harbored a rearrangement (Figure S3, Supplementary Information), where the first segment of approximately 10.4 Kb appeared to be inverted. The second segment of about 15 Kb had the same gene order as all of the other prophages.

Regarding remnant prophages (Table S4, Supplementary Information), different scenarios were observed: (i) one phage (Sw-C388-G) has lost the putative DNA primase and helicase, among other proteins of unknown function placed in the first half of the prophage genome; (ii) two phages (Sw-C520-G and Pt-259-G) most likely lost the second half of the prophage sequence; and (iii) another two phages (Is-3180-G and Pt-5303-G) most likely lost specific ORFs. Among the later, only Is-3180-G could be assembled, yielding less than 20 kb.

### Insertion Sequences

Insertion sequences (IS), comprised of two ORFs inserted into prophage genomes were found in 39.1% (9/23) of complete prophages (Table S5, Supplementary Information) classified (according to PST typing) as hpNEurope (n = 2), hpAfrica1 (n = 5) and hpEastAsia (n = 2), and in 50% (3/6) of remnant prophages classified as hpNEurope (n = 1), hpAfrica1 (n = 1) and hpSWEurope (n = 1). The complete prophages Uk-EN31-U, Uk-EN32-U, Pt-B92-G and Fr-GC43-A had IS605 inserted once in the first three cases and twice in the last case. Interestingly, in Fr-GC43-A one copy of IS605 was inverted in relation to the other copy (Figure S4, Supplementary Information). IS605 was inverted in Uk-EN31-U, Uk-EN32-U and in one of the IS of Fr-GC43-A. The prophages Pt-228_99-G, Fr-ANT170-U and Fr-MEG235-U had two copies of ISHp608. The IS was inserted in a reverse order in relation to the other copy in Fr-ANT170-U, and twice with the same orientation in Pt-228_99-G. The third IS found was IS607 in genomes Pt-1293-U and Fr-B58-M.

Concerning remnant prophages, Sw-C388-G has the IS606 inserted at its 3′ end and the second ORF is again truncated in two. Finally, Is-3180-G carries ISHp608. The remnant prophage Pt-5303-G could not be completely assembly but ISHp608 was also found in a separate contig. Despite all of our efforts, we were not able to determine if this IS was inserted into the prophage genome or not.

IS were not always found at the same position in the prophage genomes, but prophages from strains of the same country of origin tended to present the same IS at same genome context (Table S5, Supplementary Information). Nevertheless, IS were present in most cases (9/13, 69%) immediately before DNA helicase (2/9), either before or after DNA primase (4/9), after structural protein (2/9), or after holin gene (1/9), which therefore could be considered as hotspots for IS in prophages.

The transposase genes from IS605 were inserted near the lysis cassette, as described for Mu-like phages^39^, DNA helicase and DNA primase. IS607 was located adjacent to DNA primase or a structural protein and ISHp608 near DNA primase, portal protein or structural protein. In a few cases IS were inserted into a coding sequence of a structural protein (Pt-1293-U and Pt-228_99-G) or a hypothetical protein (Pt-B92-G, Fr-ANT170-U and Fr-MEG235-U), which may impinge on transcription, and the prophage genes may be non-functional. Accordingly, IS do not appear to be randomly inserted into prophage genome. Our hypothesis is that the presence of IS within the prophage genome may inactivate the lytic cycle, benefiting the host.

### Prophage insertion site

Knowledge of the insertion site of prophages provides clues about ancient acquisition and vertical heritage. Accordingly, prophages at similar loci in different genomes can derive from a single ancestral prophage^20^. Furthermore, *H. pylori* prophage insertion sites have not been extensively studied before.

Prophage insertion site was mostly conserved among *H. pylori* PST populations. Interestingly, about 50% of the prophages enrolled in the present study and especially for the populations hpAfrica1 and hpNEurope are inserted between the same two genes, S-adenosylmethionine synthetase (synthesizes S-adenosylmethionine (AdoMet)), and UDP-3-O-[3-hydroxymyristoyl] glucosamine N-acyltransferase (metabolic pathway of lipid A). These two genes are usually contiguous in the *H. pylori* genome. Prophages classified as belonging to the hpEastAsia population, although represented in a very small number, appear to be inserted between genes coding for a competence protein ComGF and a putative outer membrane protein. Phages from hpSWEurope appear to be inserted at random locations (Table 1 and S2, Supplementary Information).

The presence of tandem repeats at the 3′ end of the prophage insertion site was often verified for prophages integrated between S-adenosylmethionine synthetase and UDP-3-O-[3-hydroxymyristoyl] glucosamine N-acyltransferase (Table S6, Supplementary Information).

### Prophage phylogenetic relationships

**Figure 2:**
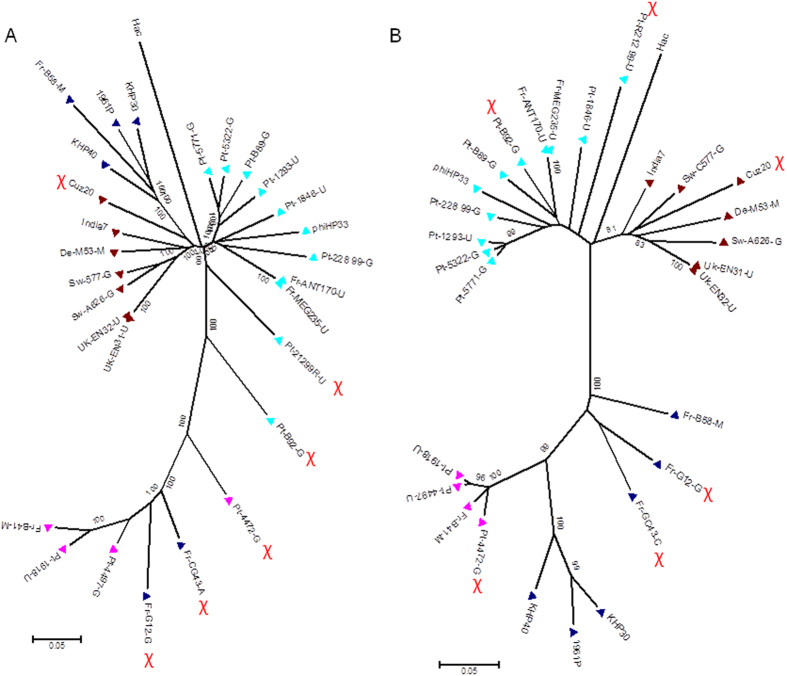
Phylogenetic trees based on (**A**) prophage genomes and (**B**) prophage sequence typing (PST). Neighbour-joining trees, Kimura two-parameter model, complete deletion option and 1000 resampling using MEGA 6.0 software. Phage population: brown triangles: hpNEurope; pink triangles: hpSWEurope; dark-blue triangles: hpEastAsia; light-blue triangles: hpAfrica1. Hac *- Helicobacter acinonychis* prophage. χ - highlights recombinogenic prophage genomes.

**Figure 3:**
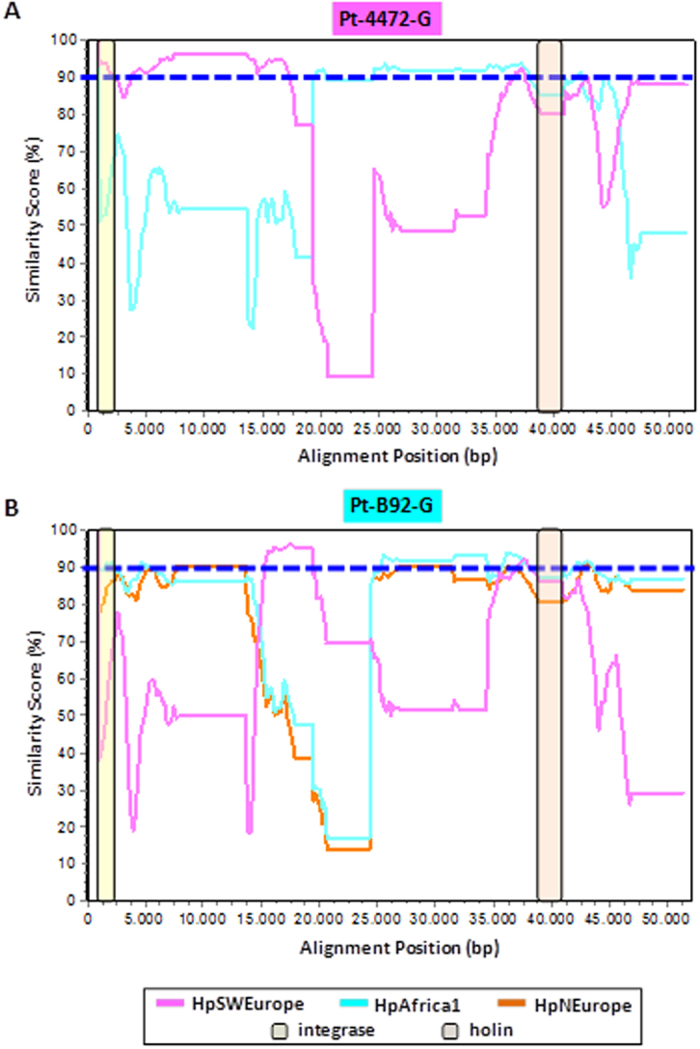
Genomic mosaicism of Pt-44772-G and Pt-B92-G prophages. (**A**) SimPlot showing the genetic similarity of PT-4472-G (PST-classified as hpSWEurope) to both the hpSWEurope and hpAfrica1 populations. (**B**) SimPlot showing the genetic similarity of Pt-B92-G (PST-classified as hpAfrica1) to hpSWEurope, hpAfrica1 and hpNEurope populations. For both plots, the Kimura 2-paramter model was used to calculate nucleotide similarities in a sliding-window of 1500 bp and a step size of 150 bp, with gap strip on. Cut-off of 90% similarity is shown in a blue dashed-line.

To get insight into the genetic backbone of the identified prophages and to infer their phylogenetic relationships in the frame of the well-known *H. pylori* geographic distribution, all 23 intact genomic sequences (Table 1) as well as the publicly available complete genomes of six *Helicobacter* phages (India7, Cuz20, 1961 P, KHP30, KHP40 and phiHP33) and the outgroup *H. acinonychis* prophage, were selected for increasing genetic diversity and were analyzed. Figure 2 shows the phylogenetic inferences found for the complete prophage genome and the concatenated integrase and holin prophage genes (PST). We observed that the majority of the prophages gather by phylogeographic group, clustering accordingly to their population assigned by STRUCTURE^40^,^41^,^42^, in a similar fashion to what we described previously for the concatenated integrase and holin genes only^33^. However, evident exceptions were noted for some prophages, namely Pt-4472-G, Fr-G12-G, Fr-CG43-A, Pt-B92-G, Pt-21299R-U and Cuz20, which displayed a discrepant phylogeographic segregation from their PST classification, suggesting the existence of putative recombination events. For instance, Pt-4472-G prophage which, according to STRUCTURE analysis, belongs to hpSWEurope, appears to be a genomic mosaic composed of both hpSWEurope and hpAfrica1 populations. This is clearly evident in Figure 3A, where Pt-4472-G is >90% similar to the latter in the genome central region, whereas the similarity to the hpSWEurope population reached values <50%. Curiously, the regions where the opposite is observed (i.e., >90% similarity to hpSWEurope) encompass both the integrase and holin genes that are used for PST classification. Another clear example of prophage recombination is exhibited by Pt-B92-G, which was PST-classified as hpAfrica1. Although most of its genome appears to be inherited from a hpAfrica1 or hpNEurope population, it displays a small middle region where similarities to the hpSWEurope population reached >95% while is strikingly different from the remainder (Figure 3B). Although less evident, we would also like to highlight two other interesting cases involving mosaicism between hpSWEurope and hpAfrica1 populations, namely Fr-G12-G and Pt-21299R-U. Despite the fact that the former was PST-classified as hpEastAsia, most of its genome was clearly inherited from a hpSWEurope population with the exception of a small 3′-end region which is highly similar to an hpAfrica1 population (data not shown). To the contrary, most of the Pt-21299R-U genome is similar to hpAfrica1, except for its 3′-end which is highly similar (>95%) to an hpSWEurope population (similarity to hpAfrica1 is as low as 40%). Interestingly, the holin gene is absent in this prophage and, in the integrase-involved region, both hpAfrica1 and hpSWEurope populations are almost equally represented (data not shown). Considering the huge genomic diversity observed among all prophage genomes, a precise identification of the location of the breakpoint regions for all of the described recombination events was not possible.

## Discussion

Most phages identified in the present study, showed a remarkable genetic synteny among themselves (Figure 1, Table S1, Supplementary Information). However, in comparison with phage KHP30, the synteny was punctuated by deletions of certain genes which were replaced by additional IS throughout the prophage genome. When prophages are present, the tendency in *H. pylori* is to have just one prophage per genome, which is in accordance with the small genome size of *H. pylori*, which is expected to have less neutral targets for prophage integration. Furthermore, *H. pylori* has slow bacterial growth, and a population at low density provides few resources for the production of virions, favoring lysogeny^21^.

Prophage ORFs were typically found in the same direction, which was opposite to that of the bacterial flanking genes. Concerning annotation most ORFs have an unknown function, as described for other species phages^43^. Although no known virulence gene was found in prophage genomes, the role of prophages in the virulence of *H. pylori* should not be immediately discarded. Frequently phages do not code for toxin genes, as they are not able of directly convert their host into a toxin producer^44^, but they can, however, indirectly modulate toxin production, such as TcdA and TcdB in *Clostridium difficille*^45^.

Considering the bacterium’s ecological niche, *H. pylori*’s persistence might be associated with both its broad genetic variability^46^ and its capability of biofilm developing^47^,^48^. In both cases the presence of extracellular DNA (eDNA) is important, either as a source of DNA taken up by the naturally competent *H. pylori*, promoting recombination or contributing to biofilm development^48^. Apart from outer membrane vesicle shedding, cell lysis via spontaneous prophage induction might be a source of eDNA release, contributing to survival and to the wide genomic variability of *H. pylori*.

The IS found in the present study were previously described in *H. pylori* but outside a prophage context^49^. IS were described to be present in about one-third of a set of 238 independent isolates of *H. pylori*^50^. Bacterial IS of IS200/IS605 and IS607 family often encode a transposase (TnpA) and a protein of unknown function, TnpB, which were hypothesized to act as a methyltransferase^51^; furthermore, *orfB* is also related to the *Salmonella* virulence gene *gipA*, a *Salmonella* prophage gene which enhances bacterial growth in Peyer’s patches^52^.

As IS found within prophage sequences showed robust homology with those found in the *H. pylori* genome, it can be hypothesized that prophages mediate the transfer of IS, further contributing to the genome plasticity of *H. pylori*. In contrast, we cannot exclude that the transfer of IS otherwise from bacteria to prophages may also be feasible. Remarkably, IS have been described in other prophages, including cyanophage Ma-LMM01, specifically-infecting *Microcystis aeruginosa* and mediating the transfer of IS607 to the bacterial genome^53^. Besides prophages, IS605 is also associated with the *cag* pathogenicity island, dividing this island into two parts called *cagI* and *cagII* by insertion of one or two copies of IS605, providing intermediate phenotypes^54^. Prophage inactivation should be under selection because lytic cycle induction may kill the cell. Correspondingly, we find five remnant prophages that might result from these evolutionary dynamics, even though defective prophages can still provide an adaptive function to bacteria^20^. Recombination with incoming phages can also imprint a signal for purifying selection. In addition, IS present in prophages have been postulated to play a role in the inactivation and immobilization of incoming phages^55^.

We showed that the prophage insertion sites can be diverse in *H. pylori* genomes although with some common traits among *H. pylori* populations, as discussed below. All prophages from hpNEurope from the present study and from *H. pylori* Cuz20 and India7 genomes (available at the NCBI), as well as most prophages from hpAfrica1 populations, have the same genomic context, presenting the bacterial genes S-adenosylmethionine synthetase and UDP-3-O-[3-hydroxymyristoyl] glucosamine N-acyltransferase at the 5′ end and 3′ end, respectively. Interestingly, the prophages genomes integrated between these two loci usually present tandem repeats at the 3′ end, between the last prophage gene and the first bacterial gene after the prophage (Table S6, Supplementary Information), most often in noncoding regions. DNA tandem repeats or satellite DNA, are inter- or intragenic nucleotide sequences repeated two or more times in a head-to-tail manner. Because these repeat tracts are prone to strand-slippage replication and recombination events causing their copy number to increase or decrease, *loci* containing tandem repeats are hypermutable^56^. Tandem repeats may reversibly shut down or modulate the function of specific genes, allowing them to adapt to changing environments on short evolutionary time scales without an increased overall mutation rate. The environmental adaptability in *H. pylori* depends primarily on tandem repeat variations, which may cause gene phase switching. DNA tandem repeats may modulate gene expression affecting transcription initiation by modifying binding affinity of regulatory proteins (upstream of −35 site) or altering the distance to promoter elements (between −35 and −10 sites), modifying the affinity of regulatory proteins or mRNA stability (between the transcriptional start and an ORF). The most frequent bacterial gene at the 5′ end of prophage codes for S-adenosylmethionine synthetase, which catalyzes the synthesis of AdoMet. AdoMet is an essential metabolic intermediate involved in many biochemical processes, such as a donor of methyl groups that allows DNA methylation (reviewed in ref. 57). Once DNA is methylated it may switch genes^6^.

All hpEastAsian prophages either described in the present study or found in the genomes of *H. pylori* YN4–84, UM038, FD430 and UM114 Asian strains (available at the NCBI) were inserted in the same genomic region, including the competence protein ComGF, which plays a role in transformation and DNA binding, at the 5′ end and a putative outer membrane protein at the 3′ end. The gene at the 5′ end is important for genetic variability of *H. pylori*^58^, while *H. pylori* outer membrane proteins are known to mediate adherence to gastric epithelium, and ultimately are associated with clinical outcome of the infection^59^. All things considered, the prophage insertion site may not be neutral for *H. pylori* gene expression and further studies are needed to evaluate the impact of prophage insertion on gene expression.

In general, the phylogenetic analysis of intact prophages presents clusters according to prophage population structure (exceptions are discussed below), confirming our previous results obtained by prophage sequence typing^33^. The prophage genomes cluster in four groups corresponding to the hpSWEurope, hpNEurope, hpAfrica1 and hpEastAsia phage populations. The strong phylogeographic signal of prophage genomes is in agreement with a model of co-evolution between the virus and its bacterial host. Indeed, prophages and bacteria are linked by a long history of co-evolution, but the genetic dimension of this co-evolution cannot be defined at present^14^. The phylogeographic clustering was in agreement with integration sites of prophages (discussed above). As suggested by others^60^, this could be explained by a vertical transmission of the phage rather than by random insertions which are common to prophages.

Phage evolution is driven by a horizontal exchange of functional modules between more or less related phages, achieved by DNA recombination, explaining the genomic mosaicism among phages^61^. Recombination is a factor of rapid variability in *H. pylori*, which is among the most recombinogenic known pathogens^12^. In parallel, in the present study, phage genomes were shown to be prone to recombination events. Indeed several prophage genome mosaics were detected, involving, for the vast majority of the cases, both hpAfrica1 and hpSWEurope populations. This is not surprising considering that both populations were detected in the same geographic area. Nevertheless, most phage ORFs are of unknown function, so no assumptions can be performed regarding a putative impact of these recombination events on pathogenicity. These mosaic structures also highlight the need for a prudent use of the PST-based classification. In fact, although an agreement is observed for most of the cases, for the studied mosaic structures, for some of the studied mosaic structures only the integrase and/or the holin genes appeared to support the PST-based classification.

The remnant prophages encountered in the present study as well as in other *H. pylori* strains^32^,^62^ and in non-*pylori Helicobacter* species^63^ highlight an evolutionary scenario consistent with a prophage decay process during the complex interaction between *H. pylori* and the prophage. However, a model in which *H. pylori* strains from different geographical regions may have been infected by distinct phage lineages after the geographic separation of the bacterial host is also feasible^11^, but less likely due to the high genetic synteny between prophages from different geographic areas. Altogether, the integration at the same locus and a gene repertoire relatedness points to a vertical transmission, suggesting the so called pervasive domestication of prophages by the bacterial host which may drive bacterial adaptation^20^. Remarkably, the most divergent *H. pylori* prophage population (hpSWEurope), presented neither conserved loci for integration site nor IS.

This work not only provides a compendium of novel sequences, but also sets the stage for future studies aimed at better understanding the virus-host relationship. Results of the present study showed that prophages are more common in *H. pylori* than initially expected and that, in most cases, prophages appear to be intact, with a sequence size of over 20 Kb. Remarkably, we show for the first time that for phages classified as hpNEurope, hpAfrica and hpEastAsia, the insertion site appears to be preserved (Table 1). Furthermore, the phylogenetic analysis for a vast majority of phage genomes is similar to the phylogenetic analysis previously presented by our team^33^ using two phage genes (integrase and holin), confirming our previous findings and reinforcing the hypothesis of co-evolution between prophages and *H. pylori*. Some recombinant phages were found, suggesting additional genetic diversity that hypothetically may provide *H. pylori* with advantageous phenotypes. Major challenges at present are to identify the function of prophage genes, to understand if the insertion site is neutral for the host and whether prophage presence plays a role in the adaptation of *H. pylori* to its host, or if prophage genes belonging to the lysis cassette are useful for biomedical applications, namely phage therapy.

## Material and Methods

### Bacteria and cell growth conditions

A total of 28 *H. pylori* strains carrying prophages were analyzed (Table S1, Supplementary Information). These included 15 strains isolated from patients with gastritis, nine from peptic ulcer patients, three from MALT patients and one from gastric cancer patient. The present study included strains from Portugal (n = 14), France (n = 6), Sweden (n = 4), UK (n = 2), Germany (n = 1) and Israel (n = 1). Prior to each assay, bacteria were grown in *H. pylori* selective medium (Biogerm, Portugal) at 37 °C in a microaerophilic environment (Anoxomat^®^, MART Microbiology BV, The Netherlands) for 24 h to 48 h. The *H. pylori* strains belong to the collection of the French National Reference Centre for Campylobacters and Helicobacters (F. Mégraud and P. Lehours, Bordeaux, France); the Department of Microbiology, Tumor and Cell Biology, Karolinska Institute (Lars Engstrand); the Klinikum Rechts Der Isar II, Medical Department, Technische Universität, Munich, Germany (M. Gerhard); the Department of Infectious Diseases, National Institute of Health, Lisbon, Portugal (M. Oleastro); and the Rabin Medical Center – Beilinson Hospital, Petah Tikva, Israel (T.T. Perets and Y. Niv).

### Whole-Genome Sequencing

Genomes were sequenced at the National Institute of Health, Lisbon, Portugal, with exception of four strains (Sw-577-G, Sw-A626-G, Sw-C388-G and Sw-C520-G) that were sequenced at Karolinska Institute, Stockholm, Sweden, and four strains (Fr-ANT170-U, Fr-MEG235-U, Fr-GC43-A and Fr-B41-M) that were sequenced at the Institute of Life Sciences, College of Medicine, Swansea, Wales, UK.

Total DNA was extracted using the QIAmp DNA Mini Kit (Qiagen, UK) according to the manufacturer’s instructions.

For genomes sequenced in Portugal and Sweden, the yield and integrity of the purified DNA were then assessed through a Qubit assay (Quanti-it dsDNA Assay Kit, Broad Range; Lifetechnologies, Paisley, CA, USA) and agarose gel electrophoresis (0.7% gel), respectively. High-quality DNA samples were then applied to prepare Nextera XT Illumina paired-end libraries. These were subsequently subjected to cluster generation and paired-end sequencing (2 × 250 bp, 2 × 150 bp and 2 × 100 bp) by using the Illumina MiSeq (Portugal) and HiSeq 2500 (Sweden) platforms (Illumina Inc., San Diego, CA, USA), according to the manufacturer’s instructions.

The number of passing filter reads obtained per sample ranged from 0.6–2.7 million reads. The FastQC (http://www.bioinformatics.babraham.ac.uk/projects/fastqc/) and FASTX (http://hannonlab.cshl.edu/fastx_toolkit/) tools were applied to evaluate and improve the quality of the raw sequence data, respectively. Subsequently, high-quality reads were *de novo* assembled using Velvet (version 1.2.10)^64^ (several assemblies using different k-mer sizes were run), where the best assembly was assumed as the one with the best cumulative ranks for N50, number of contigs/scaffolds, and length of the largest contig/scaffold. The obtained mean depth of coverage ranged from 135- to 195-fold. The final contigs/scaffolds were visually inspected (using Tablet 1.14.04.10)^65^ and corrected.

For genomes sequenced in UK, quantification of DNA was assessed after DNA extraction with a Nanodrop spectrophotometer, as well as the Quant-iT DNA Assay Kit (Life Technologies) prior to sequencing. High-throughput genome sequencing was performed using a HiSeq 2500 machine (Illumina Inc.), and the 100 bp short read paired-end data was *de novo* assembled using Velvet (version 1.2.08)^64^. The VelvetOptimiser script (version 2.2.4) was run for all odd k-mer values from 21 to 99 (several assemblies using different k-mer sizes were run), with all program settings unchanged apart from a minimum output contig size set to 200 bp and the scaffolding option switched off.

All genomes were annotated using the RAST server (http://rast.nmpdr.org/)^37^, the NCBI Prokaryotic Genomes Annotation Pipeline version 2.3. and PHAST web server^38^. The respective trimmed reads were submitted to the Sequence Read Archive (SRA).

### Assembly of prophage genomes

For prophage identification two strategies were taken. First, the PHAST web server^38^ was used to identify putative prophages within contigs of each *H. pylori* genome. Second, MEGABLAST^66^ was used to align the genome of *H. pylori* phage KHP30 or phiHP33 with the contigs of each sequenced *H. pylori* genome. PHAST analyses (http://phast.wishartlab.com/) applied over contigs allowed us to check homology, and to identify, annotate and graphically display prophage sequences, providing information on prophage completeness, categorized as either intact, incomplete, questionable or not detected. MEGABLAST was run using KHP30 or phiHP33 as reference since these prophages genomes were the most commonly found to be similar with the prophages detected by PHAST.

The MEGABLAST analysis results were particularly useful to determine which contigs were from phage origin and the order in which they probably appear. Based on this predicted contig order, primers flanking the contigs were designed, using primer3 v. 0.4.0^67^, to bridge gaps in the assembly in order to close the gaps (the gaps were of few bases to about five hundred bases). The PCR mix included Promega (Madison, WI, USA) buffer (1X), dNTPs (0.2 μM), primers (0.5 μM each), GoTaq polymerase (1.5 U), water to complete 25 μl and DNA sample (25 to 50 ng). The PCR cycle was composed of a first cycle at 95 °C for 4 min, 35 cycles at 95 °C for 30 sec, 59 °C for 30 sec and 72 °C for 1 or 2 min. A last cycle at 72 °C for 7 min was applied. The PCR products were purified using MicroSpin S-400 or S-300HR columns (GE Healthcare, Velizy-Villacoublay, France) and directly sequenced on both strands using an external sequencing service provider (Eurofins Genomics, Regensburg, Germany, and Stabvida, Lisbon, Portugal). A multiple sequence alignment^68^ was carried out using flanking parts of the contigs and the PCR sequenced product after assembly of the forward and reverse sequences.

The insertion sequences of the prophages were identified whenever the prophage 5′ and 3′ ends were contiguously flanked by bacterial genes in a contig. The last bacterial gene before the prophage sequence and the first bacterial gene after the prophage were identified as well as the homologous locus_tag for the reference genome *H. pylori* J99^36^. The presence of repeated sequences at prophage insertion sites was verified using Tandem Repeat Finder^69^ (available at https://tandem.bu.edu/trf/trf.basic.submit.html).

### Comparative genomic analyses of prophages

The assembled prophages were analyzed using PHAST to provide a first annotation. The annotation of prophage genomes was carried out further using Phages v. 1.0 (http://www.phantome.org/PhageSeed/Phage.cgi?page=phast), and RAST^37^. The annotation of coding sequences (CDS) found by the three different methods were compared.

The annotation of both *H. pylori* India7 (accession number CP002331) and Cuz20 (CP002076) prophages, as well as that of the *Helicobacter* 1961P (NC_019512.1), KHP30 (NC_019928.1), KHP40 (NC_019931.1), phiHP33 (NC_016568.1) phages, were used for comparative purposes.

The annotated prophages were aligned using the progressive Mauve algorithm software (version 2.3.1)^70^, to check the order of the CDS in the prophage genomes and the existence of a consensus sequence. In order to infer phylogenetic relationships among prophages, the intact genomes of the 23 prophages identified in the present study, were aligned using MAFFT version 7^71^ together with other six phage *Helicobacter* genomes available at public databases (1961P, KHP30, KHP40, phiHP33, *H. pylori* India7, and *H. pylori* Cuz20) as well as with the *H. acinonychis* (accession number NC_008229.1) prophage used as an outgroup. A nucleotide Neighbour-joining phylogenomic tree was constructed using the MEGA (Molecular Evolutionary Genetics Analysis) 6.0 software^72^, with distances estimated using the Kimura two-parameter model^73^. Considering the huge genomic diversity observed among all prophage genomes as well as their different lengths, both complete and pairwise deletion options were used. While the former removes all sites containing missing data or alignment gaps before the distance estimations begin, in the pairwise-deletion, option sites are only removed during the analysis as the need arises. Branching significance was estimated using bootstrap confidence levels by randomly resampling the data 1,000 times with the referred evolutionary distance model.

To determine the population structure of prophages, we use prophage sequence typing (PST), as previously described^33^. Briefly, the multi-fasta file with the alignment of integrase and holin gene sequences was converted to the STRUCTURE 2.3.4^40^,^41^,^42^ program input file using xmfa2structure by X. Didelot and D. Falush (http://www.xavierdidelot.xtreemhost.com/clonalframe.htm). STRUCTURE was used to study the number of K populations using the admixture, performing runs in duplicate. In each run, a Markov Chain Monte Carlo (MCMC) of 10,000 iterations and a burn-in period of 10,000 iterations were chosen. The highest mean value of ln likelihood was compared for multiple runs of 2 ≤ K ≤ 6.

The existence of putative recombination phenomena within prophage genomes was first evaluated using the Recombination Detection Program version 4 (RDP4)^74^ with default settings. RDP4 simultaneously applies different methods for detecting and characterizing individual recombination events that are evident within a sequence alignment without any need for predefined sets of non-recombinant reference sequences. SimPlot software (http://sray.med.som.jhmi.edu/SCRoftware/simplot/) was also used for characterizing with higher detail the genomic mosaicism of the identified recombinant prophages, as previously described for bacterial pathogens^75^. The similarity estimations were performed by using the Kimura two-parameter model with sliding window and step sizes that varied according to each recombinant genome.

### Data Availability

The genomes of the prophages are available with the accession numbers KX119174 to KX119206. The trimmed reads were submitted to the Sequence Read Archive (SRA), with the accession numbers SRP064706 to SRP064710, SRP071062, SRP071067, SRP071271, SRP071274, SRP071276 to SRP071280, SRP071282, SRP071284, SRP071289 to SRP071296, and SRP072438 to SRP072441.

## Additional Information

**How to cite this article:** Vale, F. F. *et al*. Genomic structure and insertion sites of *Helicobacter pylori* prophages from various geographical origins. *Sci. Rep.* **7**, 42471; doi: 10.1038/srep42471 (2017).

**Publisher's note:** Springer Nature remains neutral with regard to jurisdictional claims in published maps and institutional affiliations.

## Supplementary Material

Supplementary Information

## Figures and Tables

**Table 1:** Intact prophage genomes identified after whole genome sequencing.

Strain	Population		GC%		Insertion Site		Prophage				Accession number
	Phage - PST	MLST	bacteria	prophage	5′	3′	CDS* PHAST	CDS* PHAGES	CDS* RAST	Kb	
UK-EN31-U	hpNEurope	hpEurope	39.0	36.7	S-adenosylmethionine synthetase (EC 2.5.1.6) (jhp_0183)	UDP-3-O-[3-hydroxymyristoyl] glucosamine N-acyltransferase (EC 2.3.1.191) (jhp_0182)	36	34	36	30.5	KX119174
UK-EN32-U	hpNEurope	hpEurope	38.9	36.7	S-adenosylmethionine synthetase (EC 2.5.1.6) (jhp_0183)	UDP-3-O-[3-hydroxymyristoyl] glucosamine N-acyltransferase (EC 2.3.1.191) (jhp_0182)	36	34	35	29.9	KX119206
De-M53-M	hpNEurope	hpEurope	38.8	36.2	S-adenosylmethionine synthetase (jhp_0183)	UDP-3-O-[3-hydroxymyristoyl] glucosamine N-acyltransferase (EC 2.3.1.191) (jhp_0182)	33	32	33	28.1	KX119205
Sw-577-G	hpNEurope	hpEurope	38.9	36.3	S-adenosylmethionine synthetase (EC 2.5.1.6) (jhp_0183)	UDP-3-O-[3-hydroxymyristoyl] glucosamine N-acyltransferase (EC 2.3.1.191) (jhp_0182)	30	29	32	26.9	KX119204
Sw-A626-G	hpNEurope	hpEurope	38.8	36.6	ND	ND	37	32	37	31.0	KX119177
Pt-B89-G	hpAfrica1	hpEurope	39.0	37.4	S-adenosylmethionine synthetase (EC 2.5.1.6) (jhp_0183)	UDP-3-O-[3-hydroxymyristoyl] glucosamine N-acyltransferase (EC 2.3.1.191) (jhp_0182)	32	33	32	27.4	KX119203
Pt-1293-U	hpAfrica1	hpEurope	39.0	36.8	S-adenosylmethionine synthetase (EC 2.5.1.6) (jhp_0183)	UDP-3-O-[3-hydroxymyristoyl] glucosamine N-acyltransferase (EC 2.3.1.191) (jhp_0182)	36	37	36	30.1	KX119202
Fr-ANT170-U	hpAfrica1	hpEurope	39.0	37.2	S-adenosylmethionine synthetase (EC 2.5.1.6) (jhp_0183)	UDP-3-O-[3-hydroxymyristoyl] glucosamine N-acyltransferase (EC 2.3.1.191) (jhp_0182)	37	33	36	31.2	KX119201
Fr-MEG235-U	hpAfrica1	hpEurope	39.1	37.3	S-adenosylmethionine synthetase (EC 2.5.1.6) (jhp_0183)	UDP-3-O-[3-hydroxymyristoyl] glucosamine N-acyltransferase (EC 2.3.1.191) (jhp_0182)	37	33	36	31.2	KX119200
Pt-5771-G	hpAfrica1	hpEurope	39.0	36.9	S-adenosylmethionine synthetase (EC 2.5.1.6) (jhp_0183)	UDP-3-O-[3-hydroxymyristoyl] glucosamine N-acyltransferase (EC 2.3.1.191) (jhp_0182)	34	34	34	29.8	KX119199
Pt-5322-G	hpAfrica1	hpEurope	39.1	36.8	S-adenosylmethionine synthetase (EC 2.5.1.6) (jhp_0183)	UDP-3-O-[3-hydroxymyristoyl] glucosamine N-acyltransferase (EC 2.3.1.191) (jhp_0182)	31	31	31	28.3	KX119198
Pt-228_99-G	hpAfrica1	hpEurope	39.0	37.2	S-adenosylmethionine synthetase (EC 2.5.1.6) (jhp_0183)	UDP-3-O-[3-hydroxymyristoyl] glucosamine N-acyltransferase (EC 2.3.1.191) (jhp_0182)	37	36	38	30.1	KX119175
Pt-1846-U	hpAfrica1	hpEurope	39.0	37.0	GTP cyclohydrolase II/3,4-dihydroxy-2-butanone 4- phosphate synthase (jhp_0740)	ND	32	31	32	28.0	KX119176
Pt-B92-G	hpAfrica1	hpEurope	38.8	36.9	Membrane-associated phospholipid phosphatase (jhp_0787)	ND	39	36	38	30.5	KX119197
Pt-4481-G	hpAfrica1	hpEurope	39.0	36.8	ND	Ribosomal large subunit pseudouridine synthase B (EC 4.2.1.70) (jhp_1353)	32	31	32	25.4	KX119196
Fr-GC43-A	HpEastAsia	hpEurope	39.0	36.3	Competence protein ComGF (jhp_0650)	putative outer membrane protein HomA (jhp_0649)	38	37	39	33.0	KX119195
Fr-G12-G	hpEastAsia	hpEurope	38.9	36.3	Competence protein ComGF (jhp_0650)	putative outer membrane protein (jhp_0649)	36	35	36	28.6	KX119194
Fr-B58-M	hpEastAsia	hpEastAsia	38.8	36.0	Competence protein ComGF (jhp_0650)	putative outer membrane protein (jhp_0649)	26	24	26	22.6	KX119193
Pt-212-99R-U	hpAfrica1	hpEurope	38.9	37.1	Competence protein ComGF (jhp_0650)	putative outer membrane protein (jhp_0649)	24	24	24	23.0	KX119189
Pt-1918-U	hpSWEurope	hspWAfrica	39.1	36.2	Hypothetical protein (jhp_1347)	Putative outer membrane protein (jhp_1346)	34	33	34	28.7	KX119192
Pt-4497-U	hpSWEurope	hspWAfrica	39.3	36.2	hypothetical protein (jhp_0949)	Putative protein (jhp_0950)	35	34	36	29.4	KX119191
Pt-4472-G	hpSWEurope	hpEurope	38.8	36.6	hypothetical protein (jhp_0191)	hypothetical protein (jhp_0193)	32	30	32	27.6	KX119190
Fr-B41-M	hpSWEurope	hpWAfrica	39.1	35.5	Acetyl-coenzyme A carboxyl transferase alpha chain (EC 6.4.1.2) (jph_0504)	hypothetical protein (jhp_0503)	35	35	36	29.4	KX119188

^*^Number of coding sequences (CDS) detected according to web service used; GC: guanine-cytosine; PST: prophage sequence typing; MLST: multilocus sequence typing.
