# Supplementary material for: Genomic structure and insertion sites of Helicobacter pylori prophages from various geographical origins
Source: Sci Rep. 2017 Feb 16;7:42471. doi: 10.1038/srep42471 (PMC5311958; doi:10.1038/srep42471)
Supplement: Supplementary Information [file srep42471-s1.pdf]

Genomic structure and insertion sites of *Helicobacter pylori* prophages from various geographical origins

Filipa F. Vale, Alexandra Nunes, Mónica Oleastro, João P. Gomes, Daniel A. Sampaio, Raquel Rocha, Jorge M. B. Vítor, Lars Engstrand, Ben Pascoe, Elvire Berthenet, Sam Sheppard, Matthew D. Hitchings, Francis Mégraud, Jamuna Vadivelu, Philippe Lehours

Supplementary Information

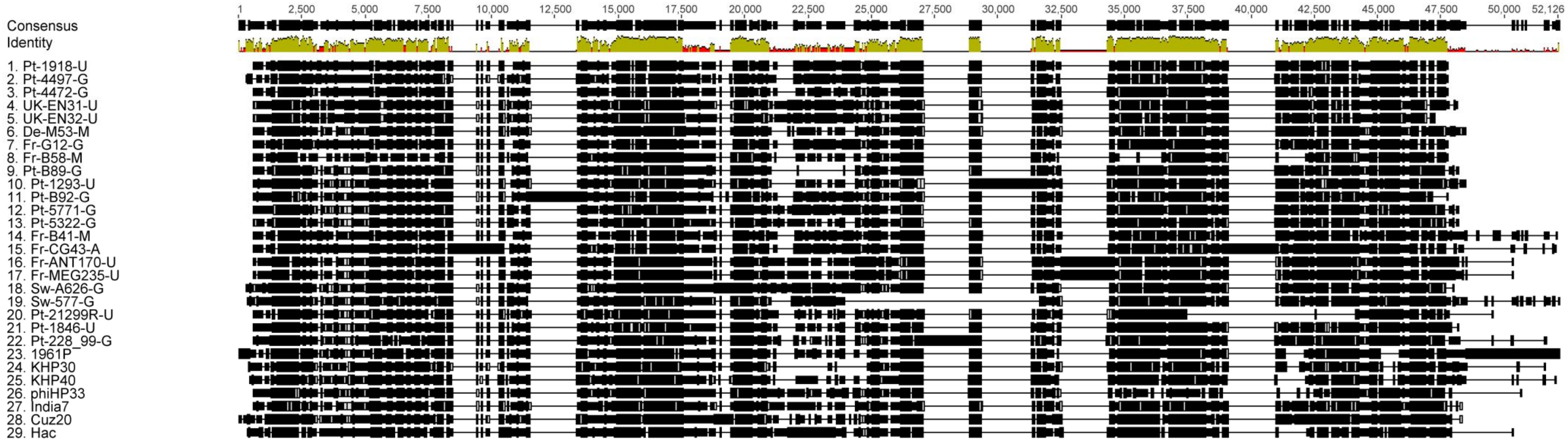

Figure S1. MAFFT alignment of prophage genomes showing consensus identity. Consensus identity along the multiple alignment of prophage genomes (bp) shows mean pairwise identity over all pairs in the column. The multiple sequence alignment is a basic tool in inferring the evolutionary history of biological sequences. For instance, the alignment of several homologs can be used to search for patterns of highly conserved regions. The detailed percent similarity between prophage genomes is presented in Figure S2. Green: 100% identity, Greenish-brown: at least 30% and under 100% identity, Red: below 30% identity.

	Pt-1918-U	Pt-4497-G	Pt-4472-G	UK-EN31-U	UK-EN32-U	De-M53-M	Fr-G12-G	Fr-B58-M	Pt-B89-G	Pt-1293-U	Pt-B92-G	Pt-5771-G	Pt-5322-G	Fr-B41-M
Pt-1918-U		91.7%	76.0%	61.2%	61.2%	59.5%	84.1%	48.2%	58.4%	55.8%	64.7%	63.0%	59.1%	90.3%
Pt-4497-G	91.7%		72.6%	63.7%	63.7%	61.9%	80.7%	49.9%	60.8%	58.0%	67.2%	65.4%	61.5%	86.3%
Pt-4472-G	76.0%	72.6%		63.9%	63.9%	67.1%	75.4%	54.4%	67.2%	62.6%	63.4%	65.8%	66.1%	75.2%
UK-EN31-U	61.2%	63.7%	63.9%		100.0%	74.9%	62.6%	56.8%	73.1%	69.0%	67.2%	79.8%	74.7%	60.2%
UK-EN32-U	61.2%	63.7%	63.9%	100.0%		74.7%	62.6%	56.6%	72.9%	68.8%	68.3%	79.8%	74.5%	60.6%
De-M53-M	59.5%	61.9%	67.1%	74.9%	74.7%		60.7%	62.3%	80.9%	76.6%	67.0%	77.3%	81.9%	57.7%
Fr-G12-G	84.1%	80.7%	75.4%	62.6%	62.6%	60.7%		48.8%	60.0%	57.0%	64.7%	64.1%	60.4%	83.0%
Fr-B58-M	48.2%	49.9%	54.4%	56.8%	56.6%	62.3%	48.8%		59.6%	57.7%	48.9%	57.3%	60.8%	47.5%
Pt-B89-G	58.4%	60.8%	67.2%	73.1%	72.9%	80.9%	60.0%	59.6%		80.3%	66.5%	80.4%	84.0%	56.7%
Pt-1293-U	55.8%	58.0%	62.6%	69.0%	68.8%	76.6%	57.0%	57.7%	80.3%		63.6%	76.4%	79.9%	54.1%
Pt-B92-G	64.7%	67.2%	63.4%	67.2%	68.3%	67.0%	64.7%	48.9%	66.5%	63.6%		70.7%	66.9%	63.3%
Pt-5771-G	63.0%	65.4%	65.8%	79.8%	79.8%	77.3%	64.1%	57.3%	80.4%	76.4%	70.7%		85.0%	61.1%
Pt-5322-G	59.1%	61.5%	66.1%	74.7%	74.5%	81.9%	60.4%	60.8%	84.0%	79.9%	66.9%	85.0%		57.5%
Fr-B41-M	90.3%	86.3%	75.2%	60.2%	60.6%	57.7%	83.0%	47.5%	56.7%	54.1%	63.3%	61.1%	57.5%	
Fr-CG43-A	73.3%	74.8%	64.4%	58.7%	58.8%	56.6%	72.5%	46.2%	54.8%	52.6%	59.7%	59.5%	55.9%	71.8%
Fr-ANT170-U	54.9%	57.0%	61.5%	67.1%	66.9%	71.1%	56.4%	53.0%	70.8%	67.9%	61.8%	71.2%	73.0%	53.0%
Fr-MEG235-U	54.9%	57.0%	61.5%	67.1%	66.9%	71.1%	56.4%	53.0%	70.8%	67.9%	61.8%	71.2%	73.0%	53.0%
Sw-A626-G	61.1%	63.7%	63.6%	81.5%	81.4%	74.6%	62.3%	55.6%	72.6%	68.7%	67.7%	80.1%	75.6%	60.3%
Sw-577-G	56.4%	59.0%	58.6%	73.4%	73.2%	71.1%	57.5%	51.9%	69.1%	65.6%	61.7%	72.6%	70.4%	54.6%
Pt-21299R-U	51.5%	53.8%	58.0%	57.6%	57.6%	63.5%	51.1%	50.9%	65.4%	61.8%	53.5%	60.7%	63.9%	50.0%
Pt-1846-U	59.4%	61.8%	67.3%	72.3%	72.2%	80.9%	60.6%	59.9%	80.5%	77.8%	67.5%	76.6%	81.7%	58.1%
Pt-228_99-G	53.8%	55.9%	62.0%	64.9%	64.7%	71.2%	55.6%	54.5%	73.3%	68.9%	61.0%	69.0%	71.8%	51.7%
1961P	56.6%	58.2%	62.7%	63.4%	63.8%	70.5%	57.5%	67.5%	68.0%	65.2%	57.2%	65.1%	69.6%	50.8%
KHP30	56.8%	58.4%	64.1%	63.7%	64.2%	70.5%	57.6%	64.5%	70.5%	64.6%	57.4%	65.4%	68.8%	56.2%
KHP40	56.1%	58.3%	63.0%	64.8%	65.3%	70.8%	56.6%	67.8%	70.4%	65.9%	57.8%	66.6%	69.8%	55.1%
phiHP33	45.7%	48.0%	51.3%	59.8%	59.4%	63.3%	46.8%	53.3%	62.9%	60.5%	53.9%	64.4%	65.1%	44.1%
India7	58.9%	61.2%	65.2%	74.6%	74.4%	82.7%	60.0%	62.0%	78.6%	75.4%	65.4%	78.9%	83.0%	57.4%
Cuz20	57.1%	58.9%	63.5%	70.8%	70.6%	76.1%	58.2%	59.0%	76.0%	70.6%	62.7%	72.0%	75.4%	55.8%
Hac	53.3%	54.9%	56.4%	63.0%	62.9%	61.9%	54.1%	51.2%	61.1%	58.1%	56.1%	64.5%	62.5%	51.7%

	Fr-CG43-A	Fr-ANT1...	Fr-MEG2...	Sw-A626-G	Sw-577-G	Pt-2129...	Pt-1846-U	Pt-228_...	1961P	KHP30	KHP40	phiHP33	India7	Cuz20	Hac
Pt-1918-U	73.3%	54.9%	54.9%	61.1%	56.4%	51.5%	59.4%	53.8%	56.6%	56.8%	56.1%	45.7%	58.9%	57.1%	53.3%
Pt-4497-G	74.8%	57.0%	57.0%	63.7%	59.0%	53.8%	61.8%	55.9%	58.2%	58.4%	58.3%	48.0%	61.2%	58.9%	54.9%
Pt-4472-G	64.4%	61.5%	61.5%	63.6%	58.6%	58.0%	67.3%	62.0%	62.7%	64.1%	63.0%	51.3%	65.2%	63.5%	56.4%
UK-EN31-U	58.7%	67.1%	67.1%	81.5%	73.4%	57.6%	72.3%	64.9%	63.4%	63.7%	64.8%	59.8%	74.6%	70.8%	63.0%
UK-EN32-U	58.8%	66.9%	66.9%	81.4%	73.2%	57.6%	72.2%	64.7%	63.8%	64.2%	65.3%	59.4%	74.4%	70.6%	62.9%
De-M53-M	56.6%	71.1%	71.1%	74.6%	71.1%	63.5%	80.9%	71.2%	70.5%	70.5%	70.8%	63.3%	82.7%	76.1%	61.9%
Fr-G12-G	72.5%	56.4%	56.4%	62.3%	57.5%	51.1%	60.6%	55.6%	57.5%	57.6%	56.6%	46.8%	60.0%	58.2%	54.1%
Fr-B58-M	46.2%	53.0%	53.0%	55.6%	51.9%	50.9%	59.9%	54.5%	67.5%	64.5%	67.8%	53.3%	62.0%	59.0%	51.2%
Pt-B89-G	54.8%	70.8%	70.8%	72.6%	69.1%	65.4%	80.5%	73.3%	68.0%	70.5%	70.4%	62.9%	78.6%	76.0%	61.1%
Pt-1293-U	52.6%	67.9%	67.9%	68.7%	65.6%	61.8%	77.8%	68.9%	65.2%	64.6%	65.9%	60.5%	75.4%	70.6%	58.1%
Pt-B92-G	59.7%	61.8%	61.8%	67.7%	61.7%	53.5%	67.5%	61.0%	57.2%	57.4%	57.8%	53.9%	65.4%	62.7%	56.1%
Pt-5771-G	59.5%	71.2%	71.2%	80.1%	72.6%	60.7%	76.6%	69.0%	65.1%	65.4%	66.6%	64.4%	78.9%	72.0%	64.5%
Pt-5322-G	55.9%	73.0%	73.0%	75.6%	70.4%	63.9%	81.7%	71.8%	69.6%	68.8%	69.8%	65.1%	83.0%	75.4%	62.5%
Fr-B41-M	71.8%	53.0%	53.0%	60.3%	54.6%	50.0%	58.1%	51.7%	50.8%	56.2%	55.1%	44.1%	57.4%	55.8%	51.7%
Fr-CG43-A		52.2%	52.2%	58.9%	53.6%	47.7%	55.9%	50.4%	48.1%	52.9%	52.5%	43.6%	56.1%	54.1%	49.8%
Fr-ANT170-U	52.2%		99.9%	67.7%	63.1%	56.9%	72.4%	67.2%	57.4%	60.9%	61.1%	60.1%	71.5%	67.1%	57.4%
Fr-MEG235-U	52.2%	99.9%		67.7%	63.1%	56.8%	72.3%	67.3%	57.4%	61.0%	61.2%	60.1%	71.5%	67.0%	57.3%
Sw-A626-G	58.9%	67.7%	67.7%		78.3%	59.6%	72.6%	65.4%	64.1%	64.3%	65.0%	60.7%	75.2%	74.6%	63.2%
Sw-577-G	53.6%	63.1%	63.1%	78.3%		54.2%	69.0%	60.5%	55.0%	61.2%	60.8%	53.3%	69.9%	67.6%	57.7%
Pt-21299R-U	47.7%	56.9%	56.8%	59.6%	54.2%		66.3%	59.0%	57.1%	59.6%	61.1%	61.8%	62.6%	63.5%	54.4%
Pt-1846-U	55.9%	72.4%	72.3%	72.6%	69.0%	66.3%		73.5%	68.8%	69.1%	69.5%	64.5%	78.3%	75.3%	62.0%
Pt-228_99-G	50.4%	67.2%	67.3%	65.4%	60.5%	59.0%	73.5%		54.7%	61.5%	61.2%	57.5%	70.0%	67.7%	56.5%
1961P	48.1%	57.4%	57.4%	64.1%	55.0%	57.1%	68.8%	54.7%		74.4%	72.7%	51.0%	69.8%	67.6%	54.2%
KHP30	52.9%	60.9%	61.0%	64.3%	61.2%	59.6%	69.1%	61.5%	74.4%		81.4%	55.1%	69.2%	69.1%	56.8%
KHP40	52.5%	61.1%	61.2%	65.0%	60.8%	61.1%	69.5%	61.2%	72.7%	81.4%		55.9%	70.6%	69.1%	58.6%
phiHP33	43.6%	60.1%	60.1%	60.7%	53.3%	61.8%	64.5%	57.5%	51.0%	55.1%	55.9%		63.5%	59.0%	54.8%
India7	56.1%	71.5%	71.5%	75.2%	69.9%	62.6%	78.3%	70.0%	69.8%	69.2%	70.6%	63.5%		75.8%	62.4%
Cuz20	54.1%	67.1%	67.0%	74.6%	67.6%	63.5%	75.3%	67.7%	67.6%	69.1%	69.1%	59.0%	75.8%		60.0%
Hac	49.8%	57.4%	57.3%	63.2%	57.7%	54.4%	62.0%	56.5%	54.2%	56.8%	58.6%	54.8%	62.4%	60.0%	

Figure S2. Heat–map representing the phage similarity matrix between 29 phages genomes based on genome content. The heat-map provides a graphical representation of data where values in a matrix are represented as a grey scale: the darker the grey, the greater the similarity.

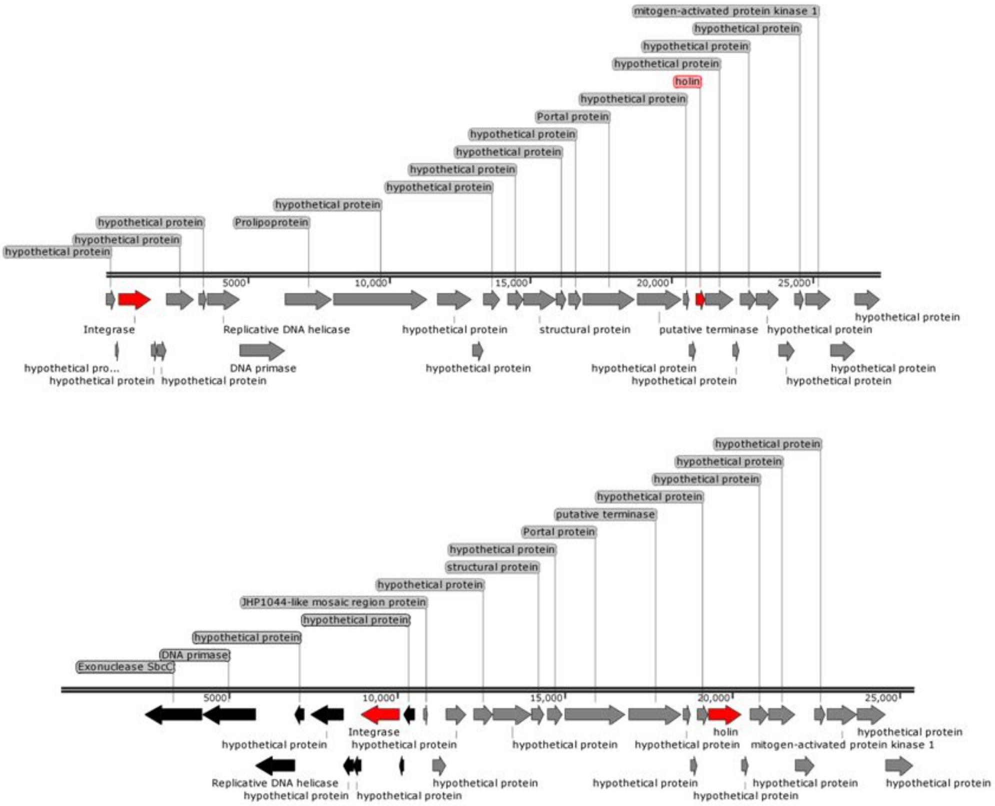

Figure S3. Genetic mapping of prophage genomes highlighting the most common positions of genes on top. Prophage open reading frame (ORF) organization for Pt-B89-G (on top) and for Pt-4481-G (on bottom). Red arrows show integrase and holin genes. Black arrows highlight an inversion of the prophage ORF for Pt-B89-G.

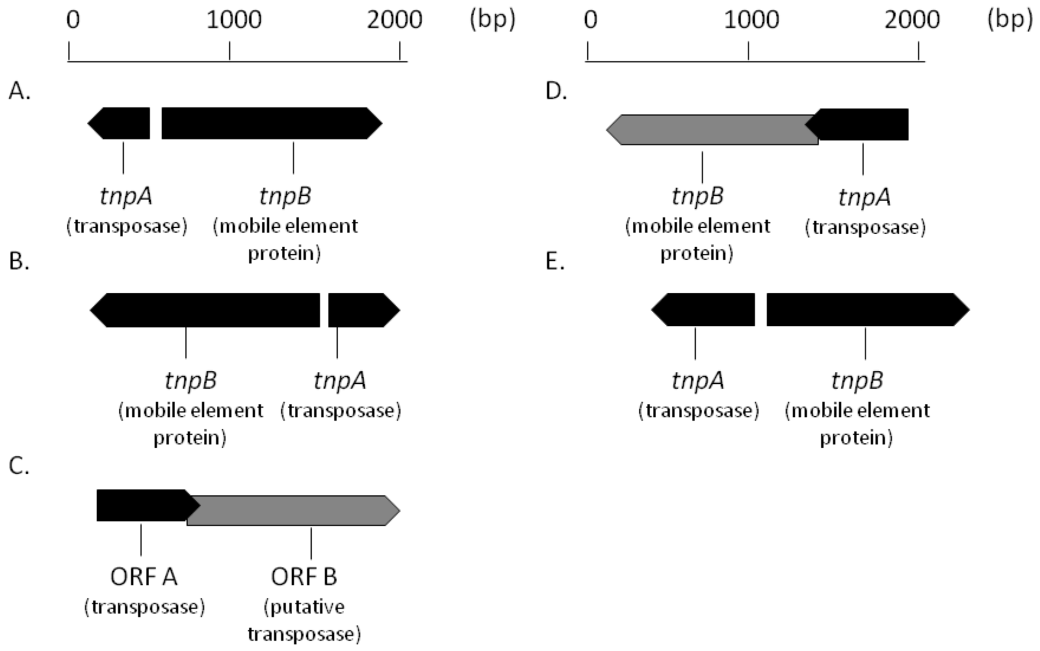

Figure S4. Genetic layout of the Insertion Sequences (IS) found in prophage genomes. A. IS605 (representation of accession number U60177) found in Pt-B92-G and in one case of Fr-GC43-A; B. IS605 found in UK-EN31-U, UK-EN32-U and in one case of Fr-GC43-A; C. ISHp608 (representation of accession number AF357224) found twice in Fr-ANT170-G and Fr-MEG235-G (copies in different sense) and once in Pt-228_99-G and Is-3180-G; D. IS607 (representation of accession number AB889602) found in Pt-1293-U and Fr-B58-M; E. IS606 (representation of accession number U95957) found in Sw-C388-G.

Table S1. Description of the 28 *H. pylori* strains that harbor prophages.

Strain	Country of isolation	Disease associated	GC%	Genome size (Mbp)	Number predicted proteins	Mean depth coverage (per base)	Bioproject accession number
UK-EN31-U	United Kingdom	Peptic Ulcer	39.0	1.61	1683	168	SRP071274
UK-EN32-U	United Kingdom	Peptic Ulcer	38.9	1.65	1694	163	SRP071276
De-M53-M	Germany	MALT lymphoma	38.8	1.65	1695	177	SRP064710
Sw-577-G	Sweden	Gastritis	38.9	1.65	1707	188	SRP071293
Sw-A626-G	Sweden	Gastritis	38.8	1.67	1709	469	SRP071294
Pt-B89-G	Portugal	Gastritis	39.0	1.62	1651	577	SRP071278
Pt-1293-U	Portugal	Peptic Ulcer	39.0	1.63	1694	202	SRP071280
Fr-ANT170-U	France	Peptic Ulcer	39.0	1.64	1663	20	SRP072438
Fr-MEG235-U	France	Peptic Ulcer	39.1	1.62	1644	20	SRP072439
Pt-5771-G	Portugal	Gastritis	39.0	1.67	1687	128	SRP064707
Pt-5322-G	Portugal	Gastritis	39.1	1.58	1621	235	SRP071284
Pt-228_99-G	Portugal	Gastritis	39.0	1.61	1652	39	SRP071067
Pt-1846-U	Portugal	Peptic Ulcer	39.0	1.54	1675	52	SRP071062
Pt-B92-G	Portugal	Gastritis	38.8	1.63	1670	261	SRP071282
Pt-4481-G	Portugal	Gastritis	39.0	1.59	1626	69	SRP071279
Fr-GC43-A	France	Gastric adenocarcinoma	39.0	1.61	1634	20	SRP072440
Fr-G12-G	France	Gastritis	38.9	1.7	1711	831	SRP064708
Fr-B58-M	France	MALT lymphoma	38.8	1.57	1630	219	SRP071277
Pt-212-99R-U	Portugal	Peptic Ulcer	38.9	1.63	1655	225	SRP071292
Pt-1918-U	Portugal	Peptic Ulcer	39.1	1.63	1663	212	SRP064706
Pt-4497-U	Portugal	Peptic Ulcer	39.3	1.61	1639	242	SRP064709
Pt-4472-G	Portugal	Gastritis	38.8	1.65	1680	343	SRP071271
Fr-B41-M	France	Gastritis	39.1	1.69	1729	20	SRP072441
Sw-C388-G	Sweden	Gastritis	38.9	1.65	1729	145	SRP071295
Sw-C520-G	Sweden	Gastritis	38.8	1.67	1698	387	SRP071296
Is-3180-G	Israel	Gastritis	39.1	1.57	1588	190	SRP071289
Pt-259-G	Portugal	Gastritis	39.0	1.59	1626	137	SRP071290
Pt-5303-G	Portugal	Gastritis	38.9	1.65	1672	245	SRP071291

Table S2. Remnant prophages identified after whole genome sequencing.

Strain	Population		GC%		Insertion Site		Prophage				Accession number
	Phage - PST	MLST	bacteria	prophage	5'	3'	CDS PHAST	CDS PHAGE	CDS RAST	Kb	
Sw-C388-G	hpNEurope	hpEurope	38.9	36.1	S-adenosylmethionine synthetase (EC 2.5.1.6) (jhp_0183)	UDP-3-O-[3-hydroxymyristoyl] glucosamine N-acyltransferase (EC 2.3.1.191) (jhp_0182)	21	21	21	13.0	KX119183
Sw-C520-G[a]	hpNEurope	hpEurope	38.8	37.7	ND	ND	14	13	13	13.6	KX119184 KX119185 KX119186 KX119187
Is-3180-G	hpAfrica1	hpEurope	39.1	37.1	S-adenosylmethionine synthetase (EC 2.5.1.6) (jhp_0183)	UDP-3-O-[3-hydroxymyristoyl] glucosamine N-acyltransferase (EC 2.3.1.191) (jhp_0182)	28	26	28	19.7	KX119182
Pt-259-G	hpSWEurope	hpEurope	39.0	36.2	hypothetical protein (jhp_0956)	hypothetical protein (jhp_0955)	12	10	11	11.6	KX119181
Pt-5303-G [b]	hpSWEurope	hpEurope	38.9	36.2	Competence protein ComGF (jhp_0650)	ND	20	21	21	19.8	KX119178 KX119179 KX119180

[a] Prophage in 4 contigs, complete genome sequence not available; [b] Prophage in 3 contigs, complete genome sequence not available. GC:guanine-cytosine; PST: prophage sequence typing; MLST: multilocus sequence typing.

Table S3. Complete prophages coding sequences.

ORF	UK-EN31-U	KHP30	UK-EN32-U	KHP30	De-M53-M	KHP30
1	hypothetical protein	G181_gp01	hypothetical protein	G181_gp01	hypothetical protein	GP181_gp01
2	Integrase	G181_gp02	Integrase	G181_gp02	hypothetical protein	
3	hypothetical protein	G181_gp03	hypothetical protein	G181_gp03	Integrase	G181_gp02
4	hypothetical protein	G181_gp04	hypothetical protein	G181_gp04	hypothetical protein	G181_gp03
5	Mobile element protein (tnpB)		Mobile element protein (tnpB)		hypothetical protein	G181_gp04
6	IS605 transposase (tnpA)		IS605 transposase (tnpA)		hypothetical protein	G181_gp05
7	DNA helicase	G181_gp07	DNA helicase	G181_gp07	hypothetical protein	G181_gp06
8	Replicative DNA helicase	G181_gp08	Replicative DNA helicase	G181_gp08	Replicative DNA helicase	G181_gp08
9	DNA primase	G181_gp09	DNA primase	G181_gp09	DNA primase	G181_gp09
10	hypothetical protein	G181_gp10	hypothetical protein	G181_gp10	Chromosome partition protein smc	G181_gp10
11	hypothetical protein	G181_gp10	hypothetical protein	G181_gp10	hypothetical protein	G181_gp11
12	hypothetical protein	G181_gp11	hypothetical protein	G181_gp11	hypothetical protein	
13	tail fiber protein		tail fiber protein		hypothetical protein	G181_gp12
14	putative tail fiber		hypothetical protein		hypothetical protein	
15	putative tail assembly protein		hypothetical protein		hypothetical protein	G181_gp13
16	hypothetical protein		hypothetical protein		structural protein	G181_gp14
17	hypothetical protein	G181_gp12	hypothetical protein	G181_gp12	hypothetical protein	G181_gp15
18	hypothetical protein	G181_gp13	hypothetical protein	G181_gp13	hypothetical protein	G181_gp16
19	structural protein	G181_gp14	structural protein	G181_gp14	Portal protein	G181_gp17
20	hypothetical protein	G181_gp15	hypothetical protein	G181_gp15	putative terminase	G181_gp18
21	hypothetical protein	G181_gp16	hypothetical protein	G181_gp16	hypothetical protein	G181_gp19
22	Portal protein	G181_gp17	Portal protein	G181_gp17	hypothetical protein	G181_gp20
23	hypothetical protein		hypothetical protein		putative holin	G181_gp21
24	putative terminase	G181_gp18	putative terminase	G181_gp18	hypothetical protein	G181_gp22
25	hypothetical protein	G181_gp19	hypothetical protein	G181_gp19	hypothetical protein	G181_gp23
26	hypothetical protein	G181_gp20	hypothetical protein	G181_gp20	hypothetical protein	G181_gp24
27	putative holin	G181_gp21	putative holin	G181_gp21	hypothetical protein	G181_gp25
28	hypothetical protein	G181_gp22	hypothetical protein	G181_gp22	hypothetical protein	G181_gp26
29	hypothetical protein	G181_gp23	hypothetical protein	G181_gp23	hypothetical protein	G181_gp27
30	hypothetical protein	G181_gp24	hypothetical protein	G181_gp24	mitogen-activated protein kinase 1	G181_gp28
31	hypothetical protein	G181_gp25	hypothetical protein	G181_gp25	hypothetical protein	G181_gp29
32	hypothetical protein	G181_gp26	hypothetical protein	G181_gp26	hypothetical protein	G181_gp29
33	hypothetical protein	G181_gp27	hypothetical protein	G181_gp27	hypothetical protein	G181_gp30
34	mitogen-activated protein kinase 1	G181_gp28	mitogen-activated protein kinase 1	G181_gp28		
35	hypothetical protein	G181_gp29	hypothetical protein	G181_gp29		
36	hypothetical protein	G181_gp30				
37						
38						
39						

Note: dark blue backgound - Coding Sequence (CDS) just annotated with RAST not with PHAST; yellow - CDS just annotated with PHAST not with RAST;
grey - phage locus_tag repeated due to the presence of a stop.

ORF	Sw-577-G	KHP30	Sw-A626-G	KHP30	Pt-B89-G	KHP30
1	hypothetical protein		hypothetical protein	G181_gp01	hypothetical protein	
2	hypothetical protein	G181_gp01	hypothetical protein		hypothetical protein	
3	hypothetical protein		Integrase	G181_gp02	Integrase	G181_gp02
4	Integrase	G181_gp02	hypothetical protein	G181_gp03	hypothetical protein	G181_gp03
5	hypothetical protein	G181_gp03	hypothetical protein	G181_gp04	hypothetical protein	G181_gp04
6	hypothetical protein	G181_gp04	hypothetical protein	G181_gp05	hypothetical protein	G181_gp05
7	hypothetical protein	G181_gp05	hypothetical protein	G181_gp06	hypothetical protein	G181_gp06
8	hypothetical protein	G181_gp06	DNA helicase	G181_gp07	Replicative DNA helicase	G181_gp08
9	DNA helicase	G181_gp07	Replicative DNA helicase	G181_gp08	DNA primase	G181_gp08
10	Replicative DNA helicase	G181_gp08	DNA primase	G181_gp09	Prolipoprotein	G181_gp10
11	DNA primase	G181_gp09	Chromosome partition protein smc	G181_gp10	hypothetical protein	G181_gp11
12	Prolipoprotein	G181_gp10	hypothetical protein		hypothetical protein	G181_gp11
13	hypothetical protein	G181_gp11	hypothetical protein		hypothetical protein	**G181_gp15**
14	hypothetical protein		tail fiber protein		hypothetical protein	G181_gp12
15	putative tail fiber		putative tail fiber		hypothetical protein	G181_gp13
16	hypothetical protein	G181_gp15	putative tail assembly protein		structural protein	G181_gp14
17	hypothetical protein	G181_gp16	hypothetical protein		hypothetical protein	G181_gp15
18	Portal protein	G181_gp17	hypothetical protein	G181_gp12	hypothetical protein	G181_gp16
19	putative terminase	G181_gp18	hypothetical protein	G181_gp13	Portal protein	G181_gp17
20	hypothetical protein	G181_gp19	structural protein	G181_gp14	putative terminase	G181_gp18
21	hypothetical protein	G181_gp20	structural protein	G181_gp14	hypothetical protein	G181_gp19
22	putative holin	G181_gp21	hypothetical protein	G181_gp15	hypothetical protein	G181_gp20
23	hypothetical protein	G181_gp22	hypothetical protein	G181_gp16	putative holin	G181_gp21
24	hypothetical protein	G181_gp23	Portal protein	G181_gp17	hypothetical protein	G181_gp22
25	hypothetical protein	G181_gp24	putative terminase	G181_gp18	hypothetical protein	G181_gp23
26	hypothetical protein	G181_gp25	hypothetical protein	G181_gp19	hypothetical protein	G181_gp24
27	hypothetical protein	G181_gp26	hypothetical protein	G181_gp20	hypothetical protein	G181_gp25
28	hypothetical protein	G181_gp27	putative holin	G181_gp21	hypothetical protein	G181_gp26
29	mitogen-activated protein kinase 1	G181_gp28	hypothetical protein	G181_gp22	hypothetical protein	G181_gp27
30	hypothetical protein	G181_gp29	hypothetical protein	G181_gp23	mitogen-activated protein kinase 1	G181_gp28
31	hypothetical protein	G181_gp30	hypothetical protein	G181_gp24	hypothetical protein	G181_gp29
32	hypothetical protein		hypothetical protein	G181_gp25	hypothetical protein	G181_gp30
33			hypothetical protein	G181_gp26		
34			hypothetical protein	G181_gp27		
35			mitogen-activated protein kinase 1	G181_gp28		
36			hypothetical protein	G181_gp29		
37			hypothetical protein	G181_gp30		
38						
39						

ORF	Pt-1293-U	KHP30	Fr-ANT170-U	KHP30	Fr-MEG235-U	KHP30
1	hypothetical protein	G181_gp01	hypothetical protein	G181_gp01	hypothetical protein	G181_gp01
2	hypothetical protein		hypothetical protein		hypothetical protein	
3	Integrase	G181_gp02	Integrase	G181_gp02	Integrase	G181_gp02
4	hypothetical protein	G181_gp03	Integrase	G181_gp02	Integrase	G181_gp02
5	hypothetical protein	G181_gp04	hypothetical protein	G181_gp03	hypothetical protein	G181_gp03
6	hypothetical protein	G181_gp05	hypothetical protein	G181_gp04	hypothetical protein	G181_gp04
7	DNA helicase	G181_gp07	hypothetical protein	G181_gp05	FIG00711007: hypothetical protein	G181_gp05
8	Replicative DNA helicase	G181_gp08	hypothetical protein	G181_gp06	hypothetical protein	G181_gp06
9	DNA primase	G181_gp09	DNA helicase	G181_gp07	DNA helicase	G181_gp07
10	DNA primase	G181_gp09	Replicative DNA helicase	G181_gp08	Replicative DNA helicase	G181_gp08
11	Exonuclease SbcC	G181_gp10	DNA primase	G181_gp09	DNA primase	G181_gp09
12	hypothetical protein	G181_gp11	Prolipoprotein	G181_gp10	Prolipoprotein	G181_gp10
13	hypothetical protein		hypothetical protein	G181_gp11	hypothetical protein	G181_gp11
14	hypothetical protein		ISHp608 transposase (OrfB)		ISHp608 transposase (OrfB)	
15	hypothetical protein	G181_gp12	Mobile element protein (OrfA)		Mobile element protein (OrfA)	
16	hypothetical protein	G181_gp13	hypothetical protein	G181_gp13	hypothetical protein	G181_gp13
17	structural protein	G181_gp14	structural protein	G181_gp14	structural protein	G181_gp14
18	Mobile element protein (tnpA)		hypothetical protein	G181_gp15	hypothetical protein	G181_gp15
19	IS607 Mobile element protein (tnpB)		hypothetical protein	G181_gp16	hypothetical protein	G181_gp16
20	structural protein	G181_gp14	Mobile element protein (OrfA)		Mobile element protein (OrfA)	
21	hypothetical protein	G181_gp15	ISHp608 transposase (OrfB)		ISHp608 transposase (OrfB)	
22	hypothetical protein	G181_gp16	hypothetical protein	G181_gp16	hypothetical protein	G181_gp16
23	Portal protein	G181_gp17	Portal protein	G181_gp17	Portal protein	G181_gp17
24	putative terminase	G181_gp18	putative terminase	G181_gp18	putative terminase	G181_gp18
25	hypothetical protein	G181_gp19	hypothetical protein		hypothetical protein	
26	hypothetical protein	G181_gp20	hypothetical protein	G181_gp20	hypothetical protein	G181_gp20
27	putative holin	G181_gp21	putative holin	G181_gp21	putative holin	G181_gp21
28	hypothetical protein	G181_gp22	hypothetical protein	G181_gp22	hypothetical protein	G181_gp22
29	hypothetical protein	G181_gp23	hypothetical protein	G181_gp23	hypothetical protein	G181_gp23
30	hypothetical protein	G181_gp24	hypothetical protein	G181_gp24	hypothetical protein	G181_gp24
31	Transcriptional regulator, MerR family	G181_gp25	hypothetical protein	G181_gp25	hypothetical protein	G181_gp25
32	hypothetical protein	G181_gp26	hypothetical protein	G181_gp26	hypothetical protein	G181_gp26
33	hypothetical protein	G181_gp27	hypothetical protein	G181_gp27	hypothetical protein	G181_gp27
34	mitogen-activated protein kinase 1	G181_gp28	mitogen-activated protein kinase 1	G181_gp28	mitogen-activated protein kinase 1	G181_gp28
35	hypothetical protein	G181_gp29	mitogen-activated protein kinase 1		mitogen-activated protein kinase 1	
36	hypothetical protein	G181_gp30	hypothetical protein	G181_gp29	hypothetical protein	G181_gp29
37			hypothetical protein	G181_gp30	hypothetical protein	G181_gp30
38						
39						

ORF	Pt-5771-G	KHP30	Pt-5322-G	KHP30	Pt-B92-G	KHP30
1	hypothetical protein	G181_gp01	hypothetical protein	G181_gp01	hypothetical protein	G181_gp01
2	hypothetical protein		hypothetical protein		hypothetical protein	
3	Integrase	G181_gp02	Integrase	G181_gp02	Integrase	G181_gp02
4	hypothetical protein	G181_gp03	hypothetical protein	G181_gp03	hypothetical protein	G181_gp03
5	hypothetical protein	G181_gp04	hypothetical protein	G181_gp04	hypothetical protein	G181_gp04
6	hypothetical protein	G181_gp05	hypothetical protein	G181_gp05	hypothetical protein	G181_gp05
7	DNA helicase	G181_gp07	DNA helicase	G181_gp07	hypothetical protein	G181_gp06
8	Replicative DNA helicase	G181_gp08	Replicative DNA helicase	G181_gp08	DNA helicase	G181_gp07
9	DNA primase	G181_gp09	DNA primase	G181_gp09	Replicative DNA helicase	G181_gp08
10	hypothetical protein	G181_gp10	hypothetical protein	G181_gp10	DNA primase	G181_gp09
11	hypothetical protein	G181_gp11	hypothetical protein	G181_gp11	hypothetical protein	G181_gp10
12	hypothetical protein		hypothetical protein		IS605 transposase (tnpA)	
13	putative tail fiber		hypothetical protein	G181_gp12	Mobile element protein (tnpB)	
14	putative tail assembly protein		hypothetical protein	G181_gp13	hypothetical protein	G181_gp10
15	hypothetical protein		structural protein	G181_gp14	hypothetical protein	G181_gp11
16	hypothetical protein	G181_gp12	hypothetical protein	G181_gp15	Tail fiber protein	
17	hypothetical protein	G181_gp13	hypothetical protein	G181_gp16	putative tail fiber	
18	structural protein	G181_gp14	Portal protein	G181_gp17	putative tail assembly protein	
19	hypothetical protein	G181_gp15	putative terminase	G181_gp18	hypothetical protein	
20	hypothetical protein	G181_gp16	hypothetical protein	G181_gp19	hypothetical protein	G181_gp12
21	Portal protein	G181_gp17	hypothetical protein	G181_gp20	hypothetical protein	G181_gp13
22	putative terminase	G181_gp18	putative holin	G181_gp21	structural protein	G181_gp14
23	hypothetical protein	G181_gp19	hypothetical protein	G181_gp22	hypothetical protein	G181_gp15
24	hypothetical protein	G181_gp20	hypothetical protein	G181_gp23	hypothetical protein	G181_gp16
25	putative holin	G181_gp21	hypothetical protein	G181_gp24	Portal protein	G181_gp17
26	hypothetical protein	G181_gp22	hypothetical protein	G181_gp25	hypothetical protein	
27	hypothetical protein	G181_gp23	hypothetical protein	G181_gp26	putative terminase	G181_gp18
28	hypothetical protein	G181_gp24	hypothetical protein	G181_gp27	hypothetical protein	G181_gp19
29	hypothetical protein	G181_gp25	mitogen-activated protein kinase 1	G181_gp28	hypothetical protein	G181_gp20
30	hypothetical protein	G181_gp26	hypothetical protein	G181_gp29	putative holin	G181_gp21
31	hypothetical protein	G181_gp27	hypothetical protein	G181_gp30	hypothetical protein	G181_gp22
32	mitogen-activated protein kinase 1	G181_gp28			hypothetical protein	G181_gp23
33	hypothetical protein	G181_gp29			hypothetical protein	G181_gp24
34	hypothetical protein	G181_gp30			hypothetical protein	G181_gp25
35					hypothetical protein	G181_gp26
36					hypothetical protein	G181_gp27
37					mitogen-activated protein kinase 1	G181_gp28
38					hypothetical protein	G181_gp29
39					putative transcriptional regulator protein	

ORF	Pt-4481-G	KHP30	Fr-GC43-G	KHP30	Fr-G12-G	KHP30
1	hypothetical protein		hypothetical protein	G181_gp01	hypothetical protein	G181_gp01
2	Exonuclease SbcC	**G181_gp10**	Integrase	G181_gp02	Integrase	G181_gp02
3	DNA primase	**G181_gp09**	hypothetical protein	G181_gp03	Integrase	G181_gp02
4	Replicative DNA helicase	**G181_gp08**	hypothetical protein	G181_gp04	hypothetical protein	G181_gp03
5	hypothetical protein	**G181_gp06**	hypothetical protein		hypothetical protein	G181_gp04
6	hypothetical protein	**G181_gp05**	hypothetical protein	G181_gp05	hypothetical protein	
7	hypothetical protein	**G181_gp04**	hypothetical protein	G181_gp06	hypothetical protein	G181_gp05
8	hypothetical protein	**G181_gp03**	putative DNA helicase	G181_gp08	hypothetical protein	G181_gp06
9	Integrase	**G181_gp02**	DNA primase	G181_gp09	hypothetical protein	
10	hypothetical protein		Mobile element protein (tnpB)		putative DNA helicase	G181_gp08
11	hypothetical protein	**G181_gp01**	IS605 transposase (tnpA)		DNA primase	G181_gp09
12	JHP1044-like mosaic region protein		Exonuclease sbcC	G181_gp10	hypothetical protein	G181_gp10
13	hypothetical protein		hypothetical protein	G181_gp11	hypothetical protein	G181_gp11
14	hypothetical protein	G181_gp12	hypothetical protein		hypothetical protein	
15	hypothetical protein		putative tail fiber		putative tail fiber	
16	hypothetical protein	G181_gp13	putative tail assembly protein		putative tail assembly protein	
17	structural protein	G181_gp14	hypothetical protein		hypothetical protein	
18	hypothetical protein	G181_gp15	hypothetical protein	G181_gp12	hypothetical protein	G181_gp12
19	hypothetical protein	G181_gp16	hypothetical protein	G181_gp13	hypothetical protein	G181_gp13
20	Portal protein	G181_gp17	structural protein	G181_gp14	structural protein	G181_gp14
21	putative terminase	G181_gp18	hypothetical protein	G181_gp15	hypothetical protein	G181_gp15
22	hypothetical protein	G181_gp19	hypothetical protein	G181_gp16	hypothetical protein	G181_gp16
23	hypothetical protein	G181_gp20	Portal protein	G181_gp17	Portal protein	G181_gp17
24	putative holin	G181_gp21	putative terminase	G181_gp18	putative terminase	G181_gp18
25	hypothetical protein	G181_gp22	hypothetical protein	G181_gp19	hypothetical protein	G181_gp19
26	hypothetical protein	G181_gp23	hypothetical protein	G181_gp20	hypothetical protein	G181_gp20
27	hypothetical protein	G181_gp24	putative holin	G181_gp21	putative holin	G181_gp21
28	hypothetical protein	G181_gp25	IS605 transposase (tnpA)		hypothetical protein	G181_gp22
29	hypothetical protein	G181_gp26	Mobile element protein (tnpB)		hypothetical protein	G181_gp23
30	hypothetical protein	G181_gp27	hypothetical protein	G181_gp22	hypothetical protein	G181_gp24
31	mitogen-activated protein kinase 1	G181_gp28	hypothetical protein	G181_gp23	hypothetical protein	G181_gp25
32	hypothetical protein	G181_gp29	hypothetical protein	G181_gp24	hypothetical protein	G181_gp26
33	hypothetical protein	G181_gp30	hypothetical protein	G181_gp25	hypothetical protein	G181_gp27
34			hypothetical protein	G181_gp26	mitogen-activated protein kinase 1	G181_gp28
35			hypothetical protein	G181_gp27	hypothetical protein	G181_gp29
36			mitogen-activated protein kinase 1	G181_gp28	hypothetical protein	G181_gp30
37			hypothetical protein	G181_gp29		
38			hypothetical protein	G181_gp30		
39			hypothetical protein			

ORF	Fr-B58-M	KHP30	Pt-1918-U	KHP30	Pt-4497-U	KHP30
1	hypothetical protein	G181_gp01	hypothetical protein	G181_gp01	hypothetical protein	
2	Integrase	G181_gp02	Integrase	G181_gp02	hypothetical protein	G181_gp01
3	First ORF in transposon ISC1904 (tnpA)		hypothetical protein	G181_gp03	Integrase	G181_gp02
4	IS607 Mobile element protein (tnpB)		hypothetical protein	G181_gp04	hypothetical protein	G181_gp03
5	DNA primase	G181_gp09	hypothetical protein		hypothetical protein	G181_gp04
6	hypothetical protein	G181_gp10	hypothetical protein	G181_gp05	hypothetical protein	
7	hypothetical protein	G181_gp11	hypothetical protein	G181_gp06	hypothetical protein	G181_gp05
8	hypothetical protein		putative DNA helicase	G181_gp08	hypothetical protein	G181_gp06
9	hypothetical protein	G181_gp12	DNA primase	G181_gp09	putative DNA helicase	G181_gp08
10	hypothetical protein	G181_gp13	hypothetical protein	G181_gp10	DNA primase	G181_gp09
11	structural protein	G181_gp14	hypothetical protein	G181_gp11	Exonuclease SbcC	G181_gp10
12	hypothetical protein	G181_gp15	tail fiber protein		hypothetical protein	G181_gp11
13	hypothetical protein	G181_gp16	putative tail fiber		hypothetical protein	
14	Portal protein	G181_gp17	putative tail assembly protein		putative tail fiber	
15	putative terminase	G181_gp18	hypothetical protein		putative tail assembly protein	
16	hypothetical protein	G181_gp19	hypothetical protein	G181_gp12	hypothetical protein	
17	hypothetical protein	G181_gp20	hypothetical protein	G181_gp13	hypothetical protein	G181_gp12
18	putative holin	G181_gp21	structural protein	G181_gp14	hypothetical protein	
19	hypothetical protein	G181_gp23	hypothetical protein	G181_gp15	hypothetical protein	G181_gp13
20	hypothetical protein	G181_gp24	hypothetical protein	G181_gp16	structural protein	G181_gp14
21	hypothetical protein	G181_gp25	Portal protein	G181_gp17	hypothetical protein	G181_gp15
22	hypothetical protein	G181_gp26	putative terminase	G181_gp18	hypothetical protein	G181_gp16
23	hypothetical protein	G181_gp27	hypothetical protein	G181_gp19	Portal protein	G181_gp17
24	mitogen-activated protein kinase 1	G181_gp28	hypothetical protein	G181_gp20	putative terminase	G181_gp18
25	hypothetical protein	G181_gp29	putative holin	G181_gp21	hypothetical protein	G181_gp19
26	hypothetical protein	G181_gp30	hypothetical protein	G181_gp22	hypothetical protein	G181_gp20
27			hypothetical protein	G181_gp23	putative holin	G181_gp21
28			hypothetical protein	G181_gp24	hypothetical protein	G181_gp22
29			hypothetical protein	G181_gp25	hypothetical protein	G181_gp23
30			hypothetical protein	G181_gp26	hypothetical protein	G181_gp24
31			hypothetical protein	G181_gp27	hypothetical protein	G181_gp25
32			mitogen-activated protein kinase 1	G181_gp28	hypothetical protein	G181_gp26
33			hypothetical protein	G181_gp29	hypothetical protein	G181_gp27
34			hypothetical protein	G181_gp30	mitogen-activated protein kinase 1	G181_gp28
35					hypothetical protein	G181_gp29
36					hypothetical protein	G181_gp30
37						
38						
39						

ORF	Pt-4472-G	KHP30	Pt-212-U	phiHP33/KHP30	Fr-B41-M	KHP30
1	hypothetical protein	G181_gp01	hypothetical protein	phiHP33_gp01	hypothetical protein	G181_gp01
2	Integrase	G181_gp02	Integrase	phiHP33_gp02	Integrase	G181_gp02
3	hypothetical protein	G181_gp03	hypothetical protein	phiHP33_gp03	hypothetical protein	G181_gp03
4	hypothetical protein	G181_gp04	putative transcriptional regulator	phiHP33_gp04	hypothetical protein	G181_gp04
5	hypothetical protein	G181_gp05	putative phage replication protein	phiHP33_gp05	hypothetical protein	
6	DNA helicase	G181_gp07	putative ABC binding casette transporter	phiHP33_gp06	hypothetical protein	G181_gp05
7	putative DNA helicase	G181_gp08	hypothetical protein	phiHP33_gp07	hypothetical protein	G181_gp06
8	putative DNA helicase	G181_gp08	Replicative DNA helicase	phiHP33_gp08	putative DNA helicase	G181_gp08
9	DNA primase	G181_gp09	DNA primase	phiHP33_gp09	DNA primase	G181_gp09
10	hypothetical protein	G181_gp10	Exonuclease SbcC	phiHP33_gp10	hypothetical protein	G181_gp10
11	hypothetical protein	G181_gp11	JHP1044 mosaic, putative crystallin beta/gamma motif-containing protein	phiHP33_gp12	hypothetical protein	G181_gp11
12	hypothetical protein	G181_gp11	hypothetical protein	phiHP33_gp14	tail fiber protein	
13	hypothetical protein		putative histidine kinase	phiHP33_gp15	putative tail fiber	
14	hypothetical protein	G181_gp12	putative histidine kinase	phiHP33_gp16	putative tail assembly protein	
15	hypothetical protein	G181_gp13	putative sensor protein	phiHP33_gp17	hypothetical protein	
16	structural protein	G181_gp14	hypothetical protein	phiHP33_gp18	hypothetical protein	G181_gp12
17	hypothetical protein	G181_gp15	hypothetical protein	phiHP33_gp19	hypothetical protein	
18	hypothetical protein	G181_gp16	Portal protein	G181_gp24	hypothetical protein	G181_gp13
19	Portal protein	G181_gp17	phage uncharacterized protein, putative		structural protein	G181_gp14
20	putative terminase	G181_gp18	putative DNA repair protein	phiHP33_gp23	hypothetical protein	G181_gp15
21	hypothetical protein	G181_gp19	hypothetical protein	phiHP33_gp24	hypothetical protein	G181_gp16
22	hypothetical protein	G181_gp20	mitogen-activated protein kinase 1	phiHP33_gp25	Portal protein	G181_gp17
23	putative holin	G181_gp21	hypothetical protein	phiHP33_gp26	putative terminase	G181_gp18
24	hypothetical protein	G181_gp22	putative transcriptional regulator protein	phiHP33_gp27	hypothetical protein	G181_gp19
25	hypothetical protein	G181_gp23			hypothetical protein	G181_gp20
26	hypothetical protein	G181_gp24			putative holin	G181_gp21
27	hypothetical protein	G181_gp25			hypothetical protein	G181_gp22
28	hypothetical protein	G181_gp26			hypothetical protein	G181_gp23
29	hypothetical protein	G181_gp27			hypothetical protein	G181_gp24
30	mitogen-activated protein kinase 1	G181_gp28			hypothetical protein	G181_gp25
31	hypothetical protein	G181_gp29			hypothetical protein	G181_gp26
32	hypothetical protein	G181_gp30			hypothetical protein	G181_gp27
33					mitogen-activated protein kinase 1	
34					hypothetical protein	G181_gp29
35					hypothetical protein	G181_gp30
36					hypothetical protein	
37						
38						
39						

ORF	Pt-1846-U	KHP30	Pt-228_99-G	KHP30
1	hypothetical protein	G181_gp01	hypothetical protein	G181_gp01
2	hypothetical protein		hypothetical protein	
3	Integrase	G181_gp02	Integrase	G181_gp02
4	hypothetical protein	G181_gp03	hypothetical protein	G181_gp03
5	hypothetical protein	G181_gp04	hypothetical protein	G181_gp04
6	hypothetical protein	G181_gp05	hypothetical protein	G181_gp05
7	hypothetical protein	G181_gp06	hypothetical protein	G181_gp06
8	DNA helicase	G181_gp07	DNA helicase	G181_gp07
9	Replicative DNA helicase	G181_gp08	Replicative DNA helicase	G181_gp08
10	DNA primase	G181_gp09	Mobile element protein (OrfA)	
11	Exonuclease sbcC	G181_gp10	ISHp608 transposase (OrfB)	
12	JHP1044 mosaic, putative crystallin beta/gamma motif-containing protein		DNA primase	G181_gp09
13	hypothetical protein		Exonuclease SbcC	G181_gp10
14	hypothetical protein	G181_gp12	hypothetical protein	G181_gp11
15	hypothetical protein	G181_gp13	hypothetical protein	
16	structural protein	G181_gp14	hypothetical protein	G181_gp12
17	hypothetical protein	G181_gp15	hypothetical protein	G181_gp13
18	hypothetical protein	G181_gp16	structural protein	G181_gp14
19	Portal protein	G181_gp17	Mobile element protein (OrfA)	
20	putative terminase	G181_gp18	ISHp608 transposase (OrfB)	
21	hypothetical protein	G181_gp19	structural protein	G181_gp14
22	hypothetical protein	G181_gp20	hypothetical protein	G181_gp15
23	putative holin	G181_gp21	hypothetical protein	G181_gp16
24	hypothetical protein	G181_gp22	Portal protein	G181_gp17
25	hypothetical protein	G181_gp23	putative terminase	G181_gp18
26	hypothetical protein	G181_gp24	hypothetical protein	G181_gp19
27	hypothetical protein	G181_gp25	hypothetical protein	G181_gp20
28	hypothetical protein	G181_gp26	putative holin	G181_gp21
29	hypothetical protein	G181_gp27	hypothetical protein	G181_gp22
30	mitogen-activated protein kinase 1	G181_gp28	hypothetical protein	G181_gp23
31	hypothetical protein	G181_gp29	hypothetical protein	G181_gp24
32	hypothetical protein	G181_gp30	hypothetical protein	G181_gp25
33			hypothetical protein	G181_gp26
34			hypothetical protein	G181_gp27
35			mitogen-activated protein kinase 1	G181_gp28
36			hypothetical protein	G181_gp29
37			hypothetical protein	G181_gp30
38			hypothetical protein	
39				

Table S4. Remnant prophages coding sequences.

ORF	Sw-C388-G	KHP30	Sw-C520-G	phiHP33	Is-3180-G	KHP30
1	hypothetical protein	G181_gp01	hypothetical protein		hypothetical protein	G181_gp01
2	hypothetical protein		hypothetical protein	phiHP33_gp01	hypothetical protein	
3	Integrase	G181_gp02	hypothetical protein		Integrase	G181_gp02
4	hypothetical protein	G181_gp03	Integrase	phiHP33_gp02	hypothetical protein	G181_gp03
5	hypothetical protein	G181_gp13	hypothetical protein	phiHP33_gp03	hypothetical protein	G181_gp04
6	structural protein	G181_gp14	putative transcriptional regulator	phiHP33_gp04	hypothetical protein	G181_gp05
7	hypothetical protein	G181_gp20	putative phage replication protein	phiHP33_gp05	hypothetical protein	G181_gp06
8	putative holin	G181_gp21	hypothetical protein	phiHP33_gp07	Replicative DNA helicase	G181_gp08
9	hypothetical protein	G181_gp22	Replicative DNA helicase	phiHP33_gp08	DNA primase	G181_gp09
10	hypothetical protein	G181_gp22	DNA primase	phiHP33_gp09	Mobile element protein (OrfA)	
11	hypothetical protein	G181_gp23	hypothetical protein	phiHP33_gp11	ISHp608 transposase (OrfB)	
12	hypothetical protein	G181_gp24	JHP1044 mosaic, putative crystallin beta/gamma motif-containing protein	phiHP33_gp12	structural protein	G181_gp14
13	hypothetical protein	G181_gp25	tail fiber protein	phiHP33_gp14	hypothetical protein	G181_gp15
14	hypothetical protein	G181_gp26	putative sensor protein	phiHP33_gp17	hypothetical protein	G181_gp16
15	hypothetical protein	G181_gp27			Portal protein	G181_gp17
16	mitogen-activated protein kinase 1	G181_gp28			putative terminase	G181_gp18
17	hypothetical protein	G181_gp29			hypothetical protein	G181_gp19
18	hypothetical protein	G181_gp30			hypothetical protein	G181_gp20
19	ISCco1, transposase orfA (IS606, tnpA)				putative holin	G181_gp21
20	Mobile element protein (IS606, tnpB)				hypothetical protein	G181_gp22
21	Mobile element protein (IS606, tnpB)				hypothetical protein	G181_gp23
22					hypothetical protein	G181_gp24
23					hypothetical protein	G181_gp25
24					hypothetical protein	G181_gp26
25					hypothetical protein	G181_gp27
26					mitogen-activated protein kinase 1	G181_gp28
27					hypothetical protein	G181_gp29
28					hypothetical protein	G181_gp30
29						
30						
31						
32						
33						
34						
35						
36						
37						
38						
39						
40						
41						
42						
43						
44						
45						
46						

Note: dark blue backgound - Coding Sequence (CDS) just annotated with RAST not with PHAST; yellow - CDS just annotated with PHAST not with RAST; codon grey - phage locus_tag repeated due to the presence of a stop.

ORF	Pt-259-G	phiHP33	Pt-5303-G	phiHP33	Fr-3842-U	KHP30
1	hypothetical protein	phiHP33_gp01	hypothetical protein		hypothetical protein	G181_gp01
2	Integrase	phiHP33_gp02	Integrase	phiHP33_gp02	Integrase	G181_gp02
3	hypothetical protein	phiHP33_gp03	hypothetical protein	phiHP33_gp03	hypothetical protein	G181_gp03
4	putative transcriptional regulator	phiHP33_gp04	hypothetical protein	phiHP33_gp04	hypothetical protein	G181_gp04
5	hypothetical protein		hypothetical protein		hypothetical protein	
6	putative phage replication protein	phiHP33_gp05	hypothetical protein	phiHP33_gp05	hypothetical protein	G181_gp05
7	putative ABC binding casette transporter	phiHP33_gp06	hypothetical protein	phiHP33_gp06	hypothetical protein	G181_gp07
8	DNA repair protein	phiHP33_gp08	DNA repair protein	phiHP33_gp08	putative DNA helicase	G181_gp08
9	hypothetical protein		DNA primase	phiHP33_gp09	DNA primase	G181_gp09
10	DNA primase	phiHP33_gp09	putative SMC (structural maintenance of chromosomes) protein	phiHP33_gp10	hypothetical protein	G181_gp10
11	Exonuclease SbcC	phiHP33_gp10	JHP1044 mosaic, putative crystallin beta/gamma motif-containing protein	phiHP33_gp12	hypothetical protein	G181_gp11
12	JHP1044 mosaic, putative crystallin beta/gamma motif-containing protein	phiHP33_gp12	tail fiber protein		hypothetical protein	G181_gp01
13			putative tail fiber		hypothetical protein	
14			putative tail assembly protein		hypothetical protein	
15			FIG00710791: hypothetical protein	phiHP33_gp14	Integrase	G181_gp02
16			putative histidine kinase	phiHP33_gp15	hypothetical protein	G181_gp03
17			putative histidine kinase	phiHP33_gp16	hypothetical protein	G181_gp04
18			Mobile element protein (OrfA)	phiHP33_gp20	hypothetical protein	G181_gp05
19			ISHp608 transposase (OrfB)	phiHP33_gp21	hypothetical protein	G181_gp06
20			hypothetical protein	phiHP33_gp26	Replicative DNA helicase	G181_gp08
21			putative transcriptional regulator protein	phiHP33_gp27	DNA primase	G181_gp09
22					Exonuclease SbcC	G181_gp10
23					hypothetical protein	G181_gp11
24					hypothetical protein	G181_gp11
25					hypothetical protein	
26					putative tail fiber	
27					putative tail assembly protein	
28					hypothetical protein	
29					hypothetical protein	G181_gp12
30					hypothetical protein	G181_gp13
31					structural protein	G181_gp14
32					hypothetical protein	G181_gp15
33					hypothetical protein	G181_gp16
34					Portal protein	G181_gp17
35					putative terminase	G181_gp18
36					hypothetical protein	G181_gp19
37					hypothetical protein	G181_gp20
38					putative holin	G181_gp21
39					hypothetical protein	G181_gp22
40					hypothetical protein	G181_gp23
41					hypothetical protein	G181_gp24
42					hypothetical protein	G181_gp25
43					hypothetical protein	G181_gp26
44					hypothetical protein	G181_gp27
45					mitogen-activated protein kinase 1	G181_gp28
46					hypothetical protein	G181_gp29

Table S5. Insertion sequences (IS) within intact prophage genomes

Prophage	IS	Copies	Insertion sense*	5' Flanking gene	3' Flanking gene
UK-EN31-U	IS605	1	Inverse	hypothetical protein	DNA helicase
UK-EN32-U	IS605	1	Inverse	hypothetical protein	DNA helicase
Fr-GC43-G	IS605	2	Inverse	DNA primase	Exonuclease sbcC
			Direct	holin	hypothetical protein
Pt-B92-G	IS605	1	Direct	DNA primase	hypothetical protein
Fr-ANT170-U	ISHp608	2	Inverse	hypothetical protein	hypothetical protein
			Direct	hypothetical protein	hypothetical protein
Fr-MEG235-U	ISHp608	2	Inverse	hypothetical protein	hypothetical protein
			Direct	hypothetical protein	hypothetical protein
Pt-228_99-G	ISHp608	2	Direct	DNA helicase	DNA primase
			Direct	structural protein	structural protein
Pt-1293-U	IS607	1	Inverse	structural protein	Structural protein
Fr-B58-M	IS607	1	Inverse	Integrase	DNA primase

* Inverse or direct sense in relation to IS605 (accession number U60177), ISHp608 (accession number AF357224) and IS607 (accession number AB889602). Details on the location of IS sequences can be found in table S3.

Table S6. Sequences of tandem repeats at the 3' end of prophage genomes.

Prophage genome	Repetition sequence at 3' end<br>Consensus pattern	Size (bp)	Copy number
De-M53-M	GGCGATGATAACGAAAGCGTGAGCGGTGTAGGTGTG<br>GTGAAGCCCTATTACCACCATGCTGTAAAAAAATTTT<br>ATATTTTAAATTTATCTTAC	36<br>37<br>20	3.1<br>4.3<br>2.4
Pt-1293-U	GCGCCTTGCCGTGCTATAAA	20	7.5
UK-EN31-U	AGCCTCCTTATAATAGCGGAAGCGTGGGCGGTGGCTATGATGTGAGCGAGTGA	53	4.9
UK-EN32-U	ATAGCGGAAGCGTGGGCGGTGGCTATGATGCGAGCGAGTGAAGCCTCCTTATA	53	5.7
Pt-B89-G	AGCGGTGGCTATGGTAGCGA	20	16.4
Pt-5771-G	GCTATAAAGCGCCTTGCCGT	20	5.8
Pt-5322-G	GCGCCTTGCCTTGCTATAAA	20	10.9
Is-3180-G	AATAAACCGCCTTGCCATTC<br>AATGTGTGTTTTT	20<br>13	4.9<br>2.8
Fr-ANT170-U	AATGTTTTCA<br>TTTTCAAATGTTTTTAAACT	10<br>20	6.8<br>3.0
Fr-MEG235-U	AATGTTTTCA<br>TTTTCAAATGTTTTTAAACT	10<br>20	6.8<br>3.0
Pt-1918-U	AATTGTGACACA	12	2.1
